# Marine algal flora of São Miguel Island, Azores

**DOI:** 10.3897/BDJ.9.e64969

**Published:** 2021-04-16

**Authors:** Ana I Azevedo Neto, Ignacio Moreu, Edgar F. Rosas Alquicira, Karla León-Cisneros, Eva Cacabelos, Andrea Z Botelho, Joana Micael, Ana C Costa, Raul M. A. Neto, José M. N. Azevedo, Sandra Monteiro, Roberto Resendes, Pedro Afonso, Afonso C. L. Prestes, Rita F. Patarra, Nuno V. Álvaro, David Milla-Figueras, Enric Ballesteros, Robert L. Fletcher, William Farnham, Ian Tittley, Manuela I. Parente

**Affiliations:** 1 cE3c - Centre for Ecology, Evolution and Environmental Changes/Azorean Biodiversity Group, Faculdade de Ciências e Tecnologia, Departamento de Biologia, Universidade dos Açores, 9500-321 Ponta Delgada, Açores, Portugal cE3c - Centre for Ecology, Evolution and Environmental Changes/Azorean Biodiversity Group, Faculdade de Ciências e Tecnologia, Departamento de Biologia, Universidade dos Açores 9500-321 Ponta Delgada, Açores Portugal; 2 Lane Community College, 4000 East 30th Ave., Eugene, Oregon, United States of America Lane Community College 4000 East 30th Ave., Eugene, Oregon United States of America; 3 Universidad Autónoma de Baja California Sur, Departamento Académico de Ciencias Marinas y Costeras, Carretera al Sur Km. 5.5, colonia el Mezquitito, La Paz, Baja California Sur, 23080, Mexico Universidad Autónoma de Baja California Sur, Departamento Académico de Ciencias Marinas y Costeras Carretera al Sur Km. 5.5, colonia el Mezquitito, La Paz, Baja California Sur, 23080 Mexico; 4 MARE – Marine and Environmental Sciences Centre, Agência Regional para o Desenvolvimento da Investigação Tecnologia e Inovação (ARDITI), Edif. Madeira Tecnopolo, Piso 2, Caminho da Penteada, Funchal, Madeira, Portugal MARE – Marine and Environmental Sciences Centre, Agência Regional para o Desenvolvimento da Investigação Tecnologia e Inovação (ARDITI) Edif. Madeira Tecnopolo, Piso 2, Caminho da Penteada, Funchal, Madeira Portugal; 5 CIBIO, Centro de Investigação em Biodiversidade e Recursos Genéticos, InBIO Laboratório Associado, Pólo dos Açores, Universidade dos Açores, Faculdade de Ciências e Tecnologia, Departamento de Biologia, 9500-321 Ponta Delgada, Açores, Portugal CIBIO, Centro de Investigação em Biodiversidade e Recursos Genéticos, InBIO Laboratório Associado, Pólo dos Açores, Universidade dos Açores, Faculdade de Ciências e Tecnologia, Departamento de Biologia 9500-321 Ponta Delgada, Açores Portugal; 6 Southwest Iceland Nature Research Centre (SINRC), Gardvegi 1, Suðurnesjabær, Iceland Southwest Iceland Nature Research Centre (SINRC) Gardvegi 1, Suðurnesjabær Iceland; 7 N/A, N/A, Portugal N/A N/A Portugal; 8 Faculdade de Ciências e Tecnologia, Departamento de Biologia, Universidade dos Açores, 9500-321 Ponta Delgada, Açores, Portugal, Portugal Faculdade de Ciências e Tecnologia, Departamento de Biologia, Universidade dos Açores 9500-321 Ponta Delgada, Açores, Portugal Portugal; 9 IMAR/Okeanos, Departamento de Oceanografia e Pescas, Universidade dos Açores, Rua Prof. Doutor Frederico Machado, 9901-862 Horta, Açores, Portugal IMAR/Okeanos, Departamento de Oceanografia e Pescas, Universidade dos Açores Rua Prof. Doutor Frederico Machado, 9901-862 Horta, Açores Portugal; 10 Expolab - Ciência Viva Science Centre, Avenida da Ciência - Beta, n.º 8, Lagoa, São Miguel, Açores, Portugal Expolab - Ciência Viva Science Centre Avenida da Ciência - Beta, n.º 8, Lagoa, São Miguel, Açores Portugal; 11 10CCMMG (Centro do Clima Meteorologia e Mudanças Globais) & IITA-A (Instituto de Investigação e Tecnologias Agrárias e do Ambiente), Universidade dos Açores, Faculdade de Ciências Agrárias, Rua Capitão João d’Ávlia – Pico da Urze, 9700-042 Angra do Heroísmo, Açores, Portugal 10CCMMG (Centro do Clima Meteorologia e Mudanças Globais) & IITA-A (Instituto de Investigação e Tecnologias Agrárias e do Ambiente), Universidade dos Açores, Faculdade de Ciências Agrárias Rua Capitão João d’Ávlia – Pico da Urze, 9700-042 Angra do Heroísmo, Açores Portugal; 12 Centre d’Estudis Avançats de Blanes-CSIC, Acc. Cala Sant Francesc 14, 17300 Blanes, Girona, Spain Centre d’Estudis Avançats de Blanes-CSIC Acc. Cala Sant Francesc 14, 17300 Blanes, Girona Spain; 13 Institute of Marine Sciences, Department of Biological Sciences, University of Portsmouth, Eastney, Portsmouth, PO4 9LY, United Kingdom Institute of Marine Sciences, Department of Biological Sciences, University of Portsmouth Eastney, Portsmouth, PO4 9LY United Kingdom; 14 Natural History Museum, Cromwell Road, London, Code SW7 5BD, United Kingdom Natural History Museum Cromwell Road, London, Code SW7 5BD United Kingdom

**Keywords:** macroalgae, Azores, São Miguel Island, new records, endemism, native, uncertain, introduced, occurrence data, ecology

## Abstract

**Background:**

The macroalgal flora of the Island of São Miguel (eastern group of the Azores Archipelago) has attracted the interest of many researchers in the past, the first publications going back to the nineteenth century. Initial studies were mainly taxonomic, resulting in the publication of a checklist of the Azorean benthic marine algae. Later, the establishment of the University of the Azores on the Island permitted the logistic conditions to develop both temporal studies and long-term research and this resulted in a significant increase on research directed at the benthic marine algae and littoral communities of the Island and consequent publications.

Prior to the present paper, the known macroalgal flora of São Miguel Island comprised around 260 species. Despite this richness, a significant amount of the research was never made public, notably Masters and PhD theses encompassing information regarding presence data recorded at littoral and sublittoral levels down to a depth of approximately 40 m around the Island and the many collections made, which resulted in vouchers deposited in the AZB Herbarium Ruy Telles Palhinha and the LSM- Molecular Systematics Laboratory at the Faculty of Sciences and Technology of the University of the Azores.

The present publication lists the macroalgal taxonomic records, together with information on their ecology and occurrence around São Miguel Island, improving the knowledge of the Azorean macroalgal flora at local and regional scales.

**New information:**

A total of 12,781 specimens (including some identified only to genus) belonging to 431 taxa of macroalgae are registered, comprising 284 Rhodophyta, 59 Chlorophyta and 88 Ochrophyta (Phaeophyceae). Of these, 323 were identified to species level (212 Rhodophyta, 48 Chlorophyta and 63 Ochrophyta), of which 61 are new records for the Island (42 Rhodophyta, 9 Chlorophyta and 10 Ochrophyta), one an Azorean endemic (Predaea feldmannii subsp. azorica Gabriel), five are Macaronesian endemisms (the red algae *Botryocladia macaronesica* Afonso-Carrillo, Sobrino, Tittley & Neto, *Laurencia viridis* Gil-Rodríguez & Haroun, *Millerella tinerfensis* (Seoane-Camba) S.M.Boo & J.M.Rico, *Phyllophora gelidioides* P.Crouan & H.Crouan ex Karsakoff and the green alga *Codium elisabethiae* O.C.Schmidt), 19 are introduced species (15 Rhodophyta, two Chlorophyta and two Ochrophyta) and 32 are of uncertain status (21 Rhodophyta, five Chlorophyta and six Ochrophyta).

## Introduction

Research on the marine algae from the Azores started in the mid-nineteenth century (1838) when Guthnick and the two Hochstetters, father and son, visited the Archipelago (Neto 1994). Since then, many other researchers and naturalists have visited the Archipelago, resulting in several publications on the marine algal flora of this region (see summary in Neto 1994, Neto 1997). Most initial studies were taxonomic, focusing on the production of species lists. Almost a century later, the German botanist Otto Christian Schmidt visited several islands, including São Miguel and initiated a more comprehensive ecological approach describing species associations and their spatial organisation (Schmidt 1931). Ever since the first half of the last century, several studies have focused more widely on intertidal and shallow subtidal communities providing information on the vertical distribution of macroalgae and invertebrates and their trophic relations (see Neto 1992, Neto 2000, Neto 2001 for a review on this subject). Taxonomic investigations have continued and the first checklist of the Azorean benthic marine algae published by Neto (1994) brought together the existing published information, provided distributional records within the Archipelago and reported 307 species, indicating a moderately rich flora given its isolated mid-Atlantic position. A revision of this first checklist was made by Parente (2010), increasing the number of algae species to 327, but without providing their distributional information on the Archipelago. Later, Rosas-Alquicira et al. (2011) published a catalogue of non-fossil geniculate coralline red algae (Corallinales, Rhodophyta) of the Macaronesia, in which they made both a critical review of species and infraspecific taxa, as well as an assessment of species diversity in the region. Research by local teams was also dedicated to the Azorean littoral communities and biota conservation (see, for example, Abecasis et al. 2015, Amorim et al. 2015, Chainho et al. 2015). Taxonomic, ecological and biotechnological investigations have continued generating knowledge on the Azorean macroalgae flora, its biotechnological potential and also on the structure and functioning of littoral communities (see revisions on Neto et al. 2014, Haroun et al. 2019 and Haroun et al. 2019). Recently, several additional studies have been published with important information on the Azorean algae biodiversity, biogeography, conservation, ecology and taxonomy (see, for example, Bruno de Sousa et al. 2019, Cacabelos et al. 2019, Cacabelos et al. 2020, Freitas et al. 2019, Kellaris et al. 2019, Martins et al. 2019, Parente et al. 2019, Parente et al. 2020, Patarra et al. 2017, Patarra et al. 2019, Patarra et al. 2020, Sousa et al. 2019, Faria et al. 2020a, Faria et al. 2020b, Vieira et al. 2020).

The paper by Freitas et al. (2019) increased the number of macroalgae species occurring in the Azores to 405 and reported that, amongst the mid-Atlantic archipelagos, the Azores is second in species richness after the Canary Islands, with 689 species, followed by Madeira (396), Cabo Verde (333) and Selvagens (295 species). For some species, the Azores Archipelago forms a boundary in their distribution. *Codium effusum* (Rafinesque) Delle Chiaje, for example, is as its western distribution limit in the Archipelago (Leon-Cisneros et al. 2012), whereas for *Dudresnaya crassa* M.Howe, a western Atlantic warm-water species, the Azores extends its known distributional range to the east. Some northern species such as the red alga *Schizymenia dubyi* (Chauvin ex Duby) J.Agardh and *Lomentaria orcadensis* (Harvey) Collins come close to their southern limit of distribution in the Azores, while some southern warm-water species, such as green alga *Anadyomene stellata* (Wulfen) C.Agardh and the red alga *Sebdenia rodrigueziana* (Feldmann) Codomier ex Athanasiadis, reach their Atlantic northern limit of distribution on the Islands (Neto et al. 2005, Leon-Cisneros et al. 2012). Some species, relatively common in the region a few years ago, have become uncommon or even very rare, for example, *Scytosiphon lomentaria* (Lyngbye) Link, *Schimmelmannia schousboei* (J.Agardh) J.Agardh. In contrast, there has been an increase in unexpected macroalgae in the Azores, with the arrival and establishment of several non-native species (see Cardigos et al. 2006, Micael et al. 2014, Vaz-Pinto et al. 2014, Parente et al. 2019, Cacabelos et al. 2019, Cacabelos et al. 2020, Martins et al. 2019).

Within the spread of the Archipelago, there are no marked differences between floras of individual Islands or Island groups and, biogeographically, the Azores algal flora reveals itself to have a mixed nature, with species shared with Macaronesia, North Africa, the Mediterranean Sea, Atlantic Europe and America (Tittley and Neto 1995, Tittley and Neto 2005, Tittley and Neto 2006, Tittley 2003, Wallenstein et al. 2009b). This nature of the Azorean marine algal flora was reinforced by the work of Freitas et al. (2019), who, using an extensive analysis encompassing data on coastal fishes, brachyurans, polychaetes, gastropods echinoderms and macroalgae, suggested that the Azores should be a biogeographical entity on its own and proposed a re-definition of the Lusitanian biogeographical province, in which they included four ecoregions: the South European Atlantic Shelf, the Saharan Upwelling area, the Azores ecoregion and a new ecoregion they named Webbnesia, which comprises the archipelagos of Madeira, Selvagens and the Canary Islands.

Not all the Azorean Islands have received the same attention regarding the studies on macroalgae. Furthermore, many species may have been overlooked due to their small size, opportunistic nature or ephemeral life span.

To overcome this and gain a better and up-to-date knowledge of the Archipelago’s macroalgae flora, an effort was made by resident teams to undertake a considerable amount of research over the past three decades on several Islands. The present paper is the last one of a series and presents physical, occurrence data and information gathered from macroalgal surveys undertaken on São Miguel Island between 1989 and 2019 mainly by the Island Aquatic Research Group of the Azorean Biodiversity Centre of the University of the Azores (Link: https://ce3c.ciencias.ulisboa.pt/sub-team/island-aquatic-ecology), the BIOISLE, Biodiversity and Islands Research Group of CIBIO-Açores at the University of the Azores (Link: https://cibio.up.pt/research-groups-1/details/bioisle) and the OKEANOS Centre of the University of the Azores (Link: http://www.okeanos.uac.pt). In these surveys, particular attention was given to the small filamentous and thin sheet-like forms that are often short-lived and fast-growing and usually very difficult to identify in the field, without the aid of a microscope and specialised literature in the laboratory.

This paper aims to provide a valuable marine biological tool to aid research on the systematics, diversity and conservation, biological monitoring, climate change, ecology and more applied studies, such as biotechnological applications, which will be of assistance to a wide range of focal groups including academics, students, governments, private organisations and the general public.

## General description

### Purpose

This paper presents taxonomic records of macroalgae for São Miguel Island and provides general information on their occurrence and distribution. By doing this, it will contribute to address several biodiversity shortfalls (see Cardoso et al. 2011, Hortal et al. 2015), namely the need to catalogue the Azorean macroalgae (Linnean shortfall) to improve current information on their local and regional geographic distribution (Wallacean shortfall), as well as to provide a better understanding of species abundance and dynamics in space (Prestonian shortfall).

## Project description

### Title

Marine algal flora of São Miguel Island, Azores

### Personnel

Collections were conducted and occurrence data recorded over several years (1989 - 2019). The main collectors were Adriá Pajares, Afonso C. L. Prestes, Alexandra Pacheco, Amine Sebti, Ana Bettencourt, Ana Carreiro, Ana Cristina Costa, Ana F. Ferreira, Ana Filipa Sousa, Ana I. Neto, Ana Leonado, Ana Rita Carreiro, Ana Rodriguez, André Amaral, André Gillon, Andrea Salamanca, Andrea Z. Botelho, Andreia Levi, Andreia Tracana, Anna Lloveras, Antalova Janouchová, Artur Oliveira, Brigida Garcia, Bruno Magalhães, Bruno Sérgio, Camille Fontaine, Carlos Campos, Carlos Mir, Carlos Rius, Carolina Moreira, Catarina Santos, Célia Albuquerque, Clara Gaspar, Cláudia Hipólito, Cristiana Figueredo, Cristina Seijo, Dálida Pereira, Daniel Torrão, Daniela Gabriel, David Milla-Figueras, Délia Cravo, Dinis Geraldes, Dolores Campos, Edgar F. Rosas-Alquicira, Emanuel Xavier, Enric Ballesteros, Eunice Nogueira, Eva Cacabelos, Fernando Feiteira, Filipe Parreira, Flávio Rodrigues, Francisco Wallenstein, Gloria Cantos, Gustavo Martins, Heather Baldwin, Helena Abreu, Hélio Dias, Hugo Lopes, Ian Tittley, Ignacio Moreu, Isadora Moniz, Joana Duarte, Joana Matzen, Jana Verdura, Joana Michael, João Brum, João Faria, João Feijó, José M. N. Azevedo, José Medeiros, Juan Garcia Marino, Juan Izaguirre, Juliana Dal Molin, Juliane Bernardi, Karla León-Cisneros, Laura Rovira, M. Canto, Marco Enoch, Margarida Leonardo, Manuela I. Parente, Marc Balcells, Marc Fernandez, Marco Henrique, Marco Santos, Maria Ana Dionísio, Maria Inês Pavão, Maria Machín-Sánchez, Maria Vale, Mariano Rego, Marisa Toste, Marlene Terra, Marta Coca, Miguel Frada, Mikel Mendizabal, Miguel Furtado, Miguel Matias, Miriam Gutierrez, Mutue Toyota Fujii, Natália Jardim, Nikola Zic, Nil Alvarez Segura, Nuno Vaz Álvaro, Núria Vila, O. Laclaustra, Olaia Morán, Olalla Torrontegi, Olivie Laroche, Patrícia Madeira, Patrícia Pereira, Paula Avelar, Paulo Azevedo, Paulo Custódio, Paulo Torres, Pedro Cavazin, Pedro Cerqueira, Pedro Raposeiro, Pedro Rodrigues, Rafael Fraga, Raquel Torres, Renato Calado, Ricardo Lacerda, Rita F. Patarra, Rita Grilo, Rita Norberto, Robert Fletcher, Rocio Sanchez, Roger Fuste, Ruben Couto, Rui Costa, Rui Jesus, Rui Moreira, Rui Patrício, Rui Sousa, Sabrina Garcia, Sandra Monteiro, Sara Peres, Sérgio Ávila, Silvia Escarduça, Sofia Carreiro, Susan Clayden, Tarso Costa, Valeria Cassano and William Farnham.

Preliminary in situ identifications were undertaken by: Ana I Neto, Andrea Z. Botelho, Andreia Levi, Daniela Gabriel, David Milla-Figueras, Edgar F. Rosas-Alquicira, Enric Ballesteros, Eva Cacabelos, Francisco Wallenstein, Heather Baldwin, Ian Tittley, Ignacio Moreu, Karla León-Cisneros, Manuela I. Parente, Maria Machín-Sanchez, Marlene Terra, Mutue Toyota Fujii, Nuno Vaz Álvaro, Raquel Torres, Robert Fletcher, Ruben Couto, Valeria Cassano and William Farnham.

Final species identification were undertaken by Ana I. Neto, Daniela Gabriel, Edgar F. Rosas-Alquicira, Enric Ballesteros, Eva Cacabelos, Ian Tittley, Ignacio Moreu, Karla León-Cisneros, Manuela I. Parente, Maria Machín-Sanchez, Marlene Terra, Mutue Toyota Fujii, Robert Fletcher, Valeria Cassano and William Farnham.

Voucher specimen management was mainly undertaken by Afonso C.L. Prestes, Ana I. Neto, Eunice Nogueira, Manuela I. Parente, Natália Cabral, Rita Patarra and Roberto Resendes.

### Study area description

**Figure 1.**
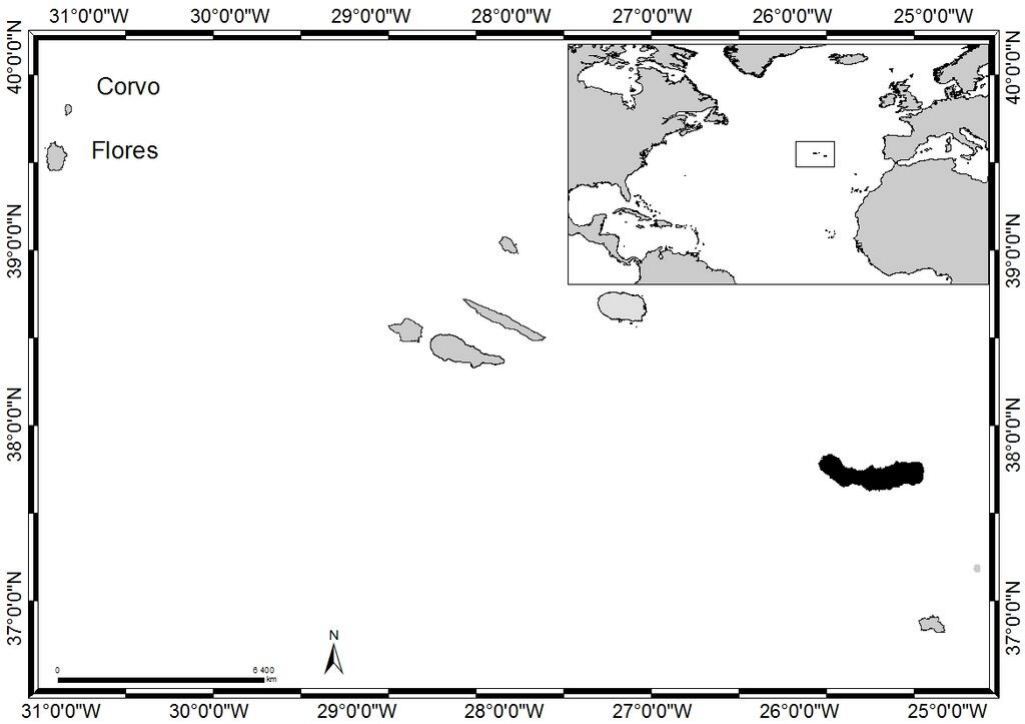
The Azores, its location in the Atlantic and São Miguel Island highlighted in black (by Nuno V. Álvaro).

The Azores Archipelago (38°43′49″N, 27°19′10″W, Fig. 1), isolated in the mid-Atlantic Ocean, comprises nine volcanic Islands and several islets spread over 500 km in a WNW–ESE direction, emerging from the Azores Plateau and located above an active triple junction between three of the world's largest tectonic plates (the North American Plate, the Eurasian Plate and the African Plate, Hildenbrand et al. 2014).

The Archipelago comprises nine volcanic Islands and several small Islets in three separate groups (eastern, central and western).

**Figure 2.**
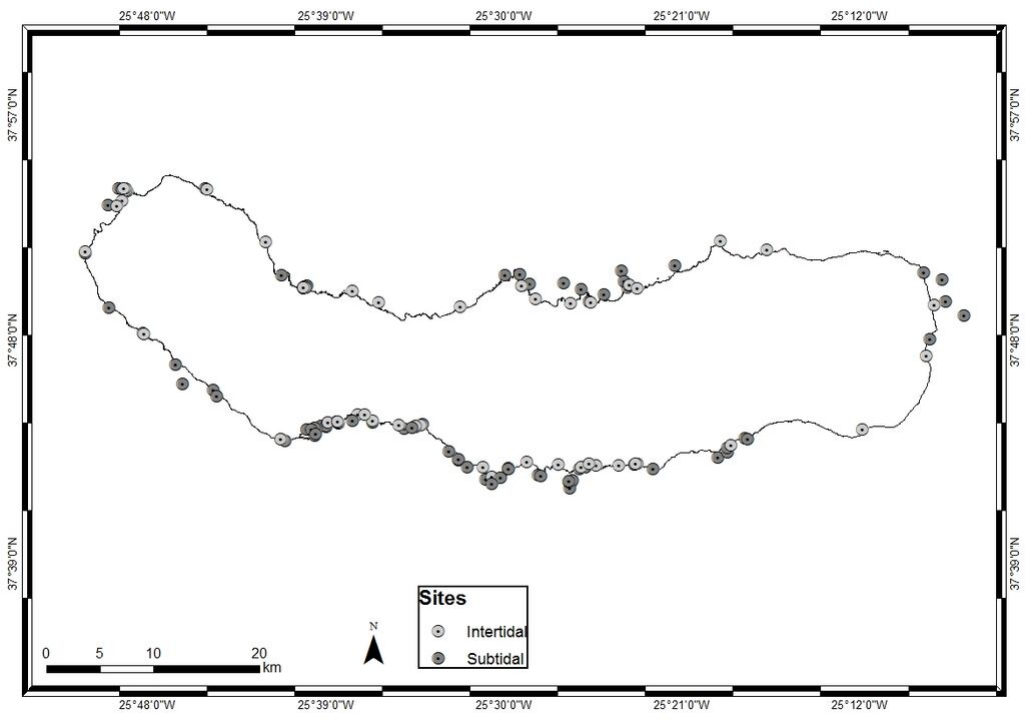
São Miguel Island with indication of the sampling locations (by Nuno V. Álvaro).

São Miguel (in black in Fig. 1), approximately 750 km² in size, is the largest and most volcanically-active Island (Gaspar et al. 2015). Located in the eastern group of the Archipelago (37°54'58''N, -25°51'52''W, Fig. 2), its formation followed a series of volcanic events, with different parts of the Island having different ages. The oldest portion (4 M years old) is the eastern side, Nordeste, where Pico da Vara (the highest mountain of the Island with 1103 m a.s.l.) is located. The Island was then progressively formed to the west: Povoação (2 M years); Furnas (750,000 years); Serra de Água de Pau (250,000 years). The Sete Cidades complex appeared 500,000 years ago and only later (50,000 years ago) was connected to Serra de Agua de Pau through the Serra Gorda and its succeeding line of peaks (Zbyszewski et al. 1958, Zbyszewski and Ferreira 1959).

As in the other Azorean Islands, the climate is considerably influenced by the surrounding ocean and is characterised by regular rainfall, medium levels of relative humidity and persistent winds, mainly during the winter and autumn seasons (Morton et al. 1998). The tidal range is small (< 2 m) and the coastal extension is restricted, with deep waters occurring within a few kilometres offshore (Hidrográfico 1981). Most sea-shores are subject to swell and surge most of the year and few are sheltered, except for some bays and harbours. Extremely heavy seas occur during winter (Neto et al. 2005).

São Miguel has the longest coastline in the archipelago, about 155 km, corresponding to 25.3% of the whole Azorean coastline. The coastal topology, resulting from the effect of the maritime agitation, responsible for the predominance of erosive morphologies, is mainly composed of high, steep cliffs with a variety of stack, arch and gully formations and is mostly difficult to access by land. Most of the cliffs and coastal slopes are less than 50 m a.s.l. (Borges 2003) and fall directly into the sea. The coastline is mainly composed of irregular compact, bedrock platforms, alternating with boulder and cobble locations. On some shores, boulders entrap coarse sand and gravel and there are a few sandy beaches (Wallenstein et al. 2009b).

**Figure 3.**
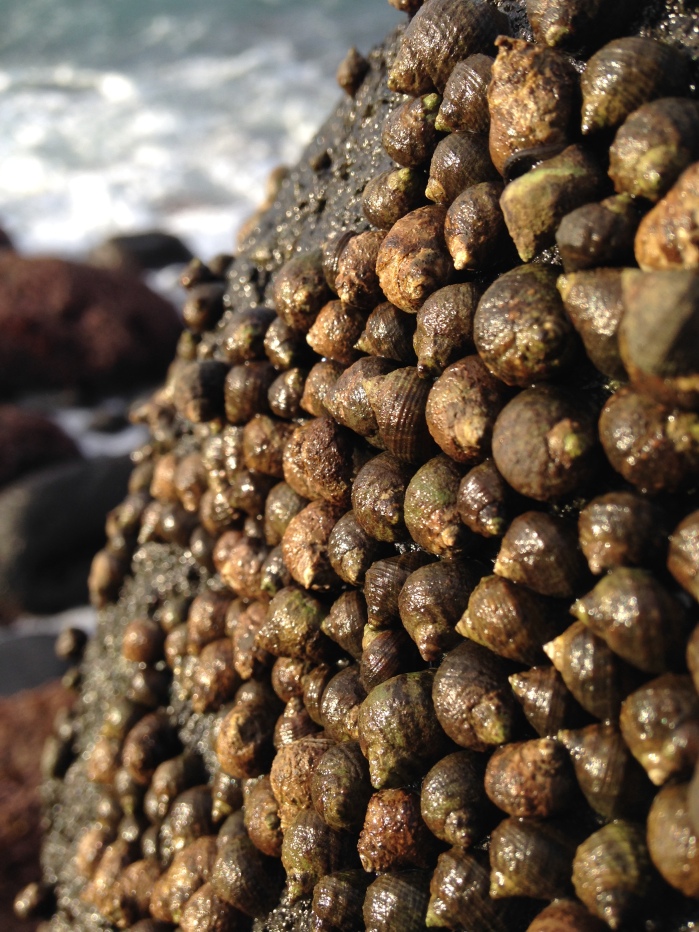
Littorinids, a characteristic gastropod species of the Azorean high intertidal level (by the Island Aquatic Ecology Subgroup of cE3c-ABG).

**Figure 4.**
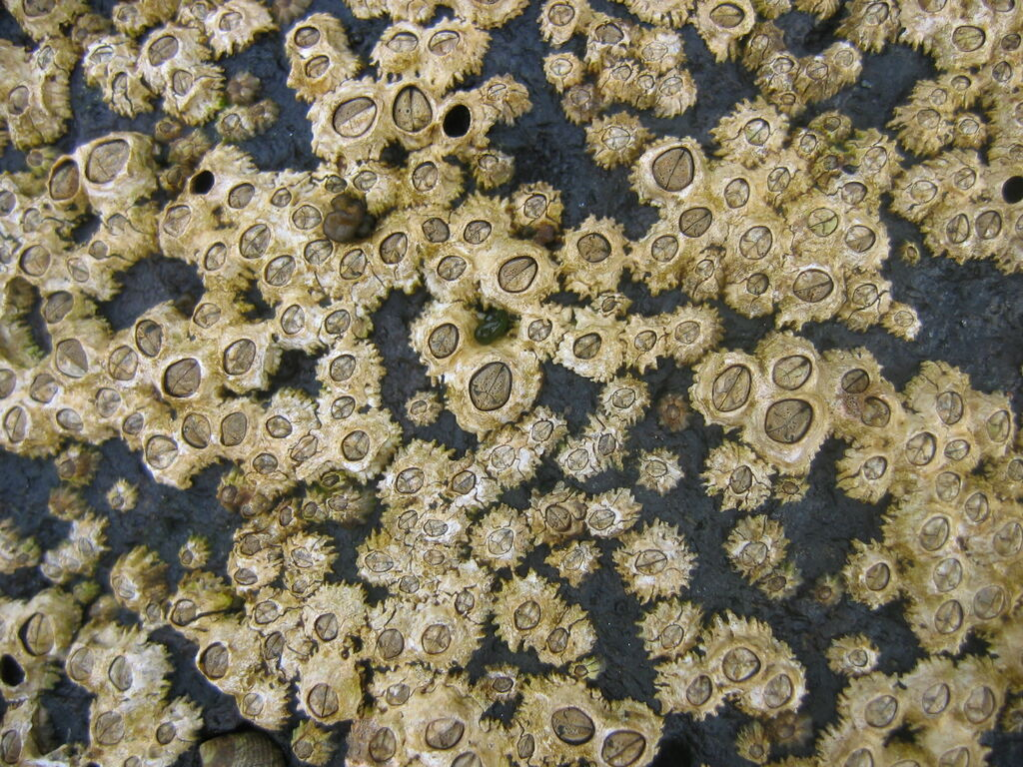
Chthamalid barnacles on São Miguel mid-intertidal level (by the Island Aquatic Ecology Subgroup of cE3c-ABG).

**Figure 5.**
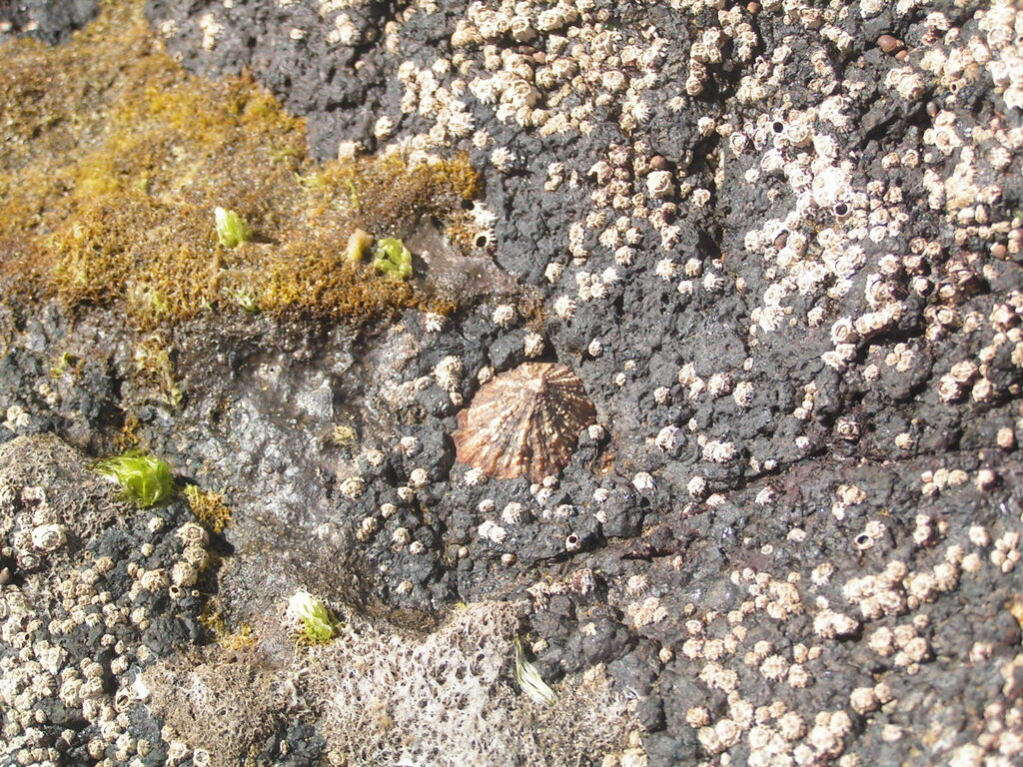
Chthamalid barnacles, algal turf and limpet on São Miguel mid-intertidal level (by the Island Aquatic Ecology Subgroup of cE3c-ABG).

**Figure 6.**
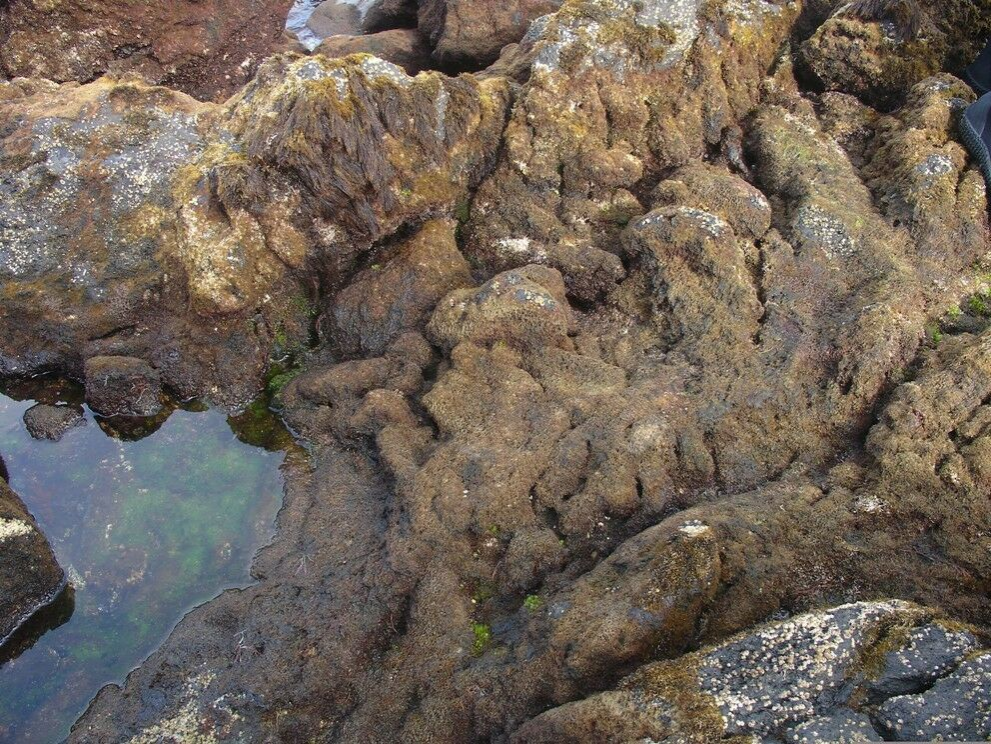
Algal turf, dominated by the red alga *Caulacanthus ustulatus*, on São Miguel mid-intertidal level (by the Island Aquatic Ecology Subgroup of cE3c-ABG).

**Figure 7.**
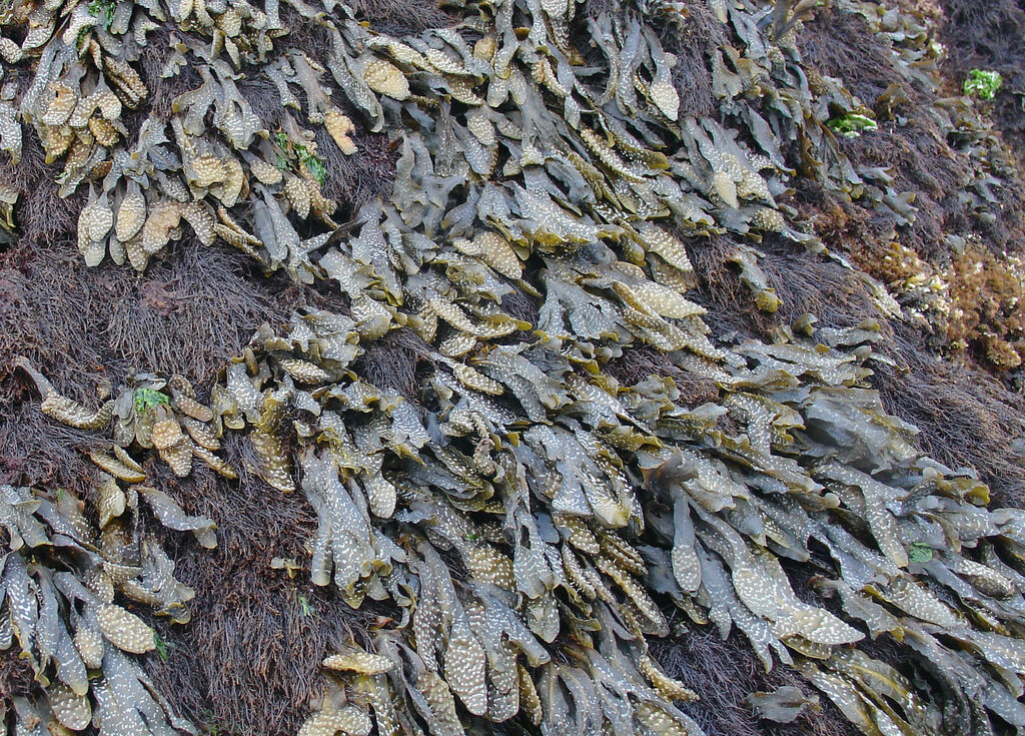
The brown alga *Fucus spiralis* and the red agarophyte *Gelidium microdon* on São Miguel mid-intertidal level (by the Island Aquatic Ecology Subgroup of cE3c-ABG).

**Figure 8.**
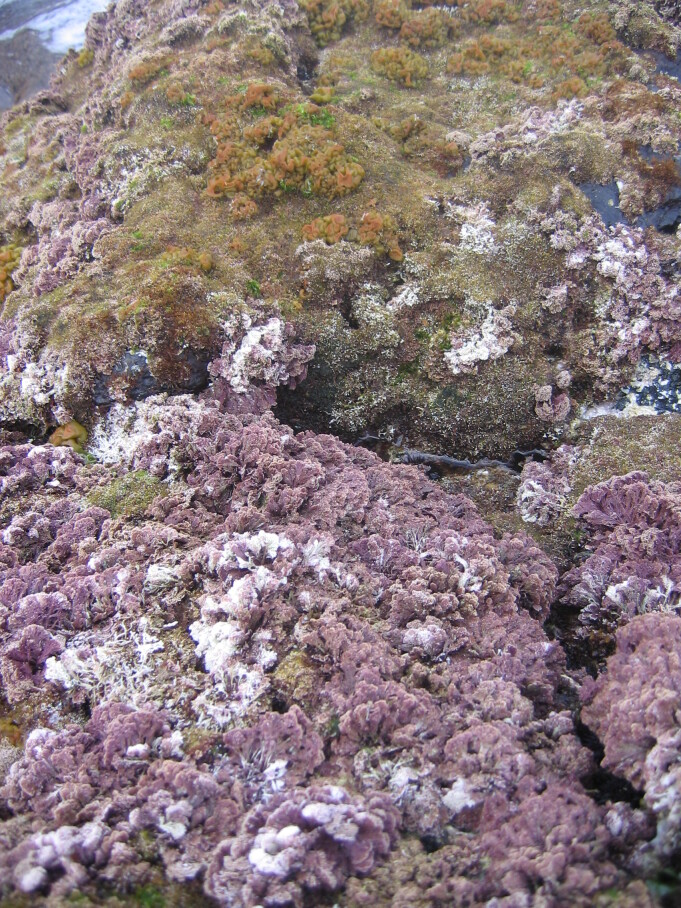
Multispecific algal turf and the coralline red alga *Ellisolandia elongata* on São Miguel low intertidal level (by the Island Aquatic Ecology Subgroup of cE3c-ABG).

**Figure 9.**
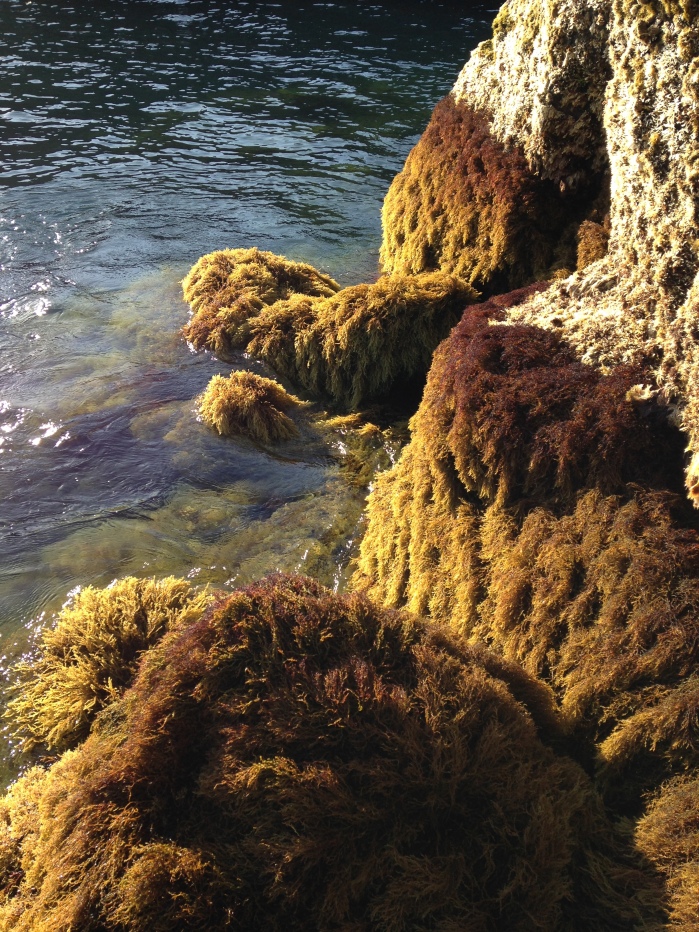
The brown alga *Gongolaria abies-marina* growing in bands at the low shore level (by the Island Aquatic Ecology Subgroup of cE3c-ABG).

**Figure 10.**
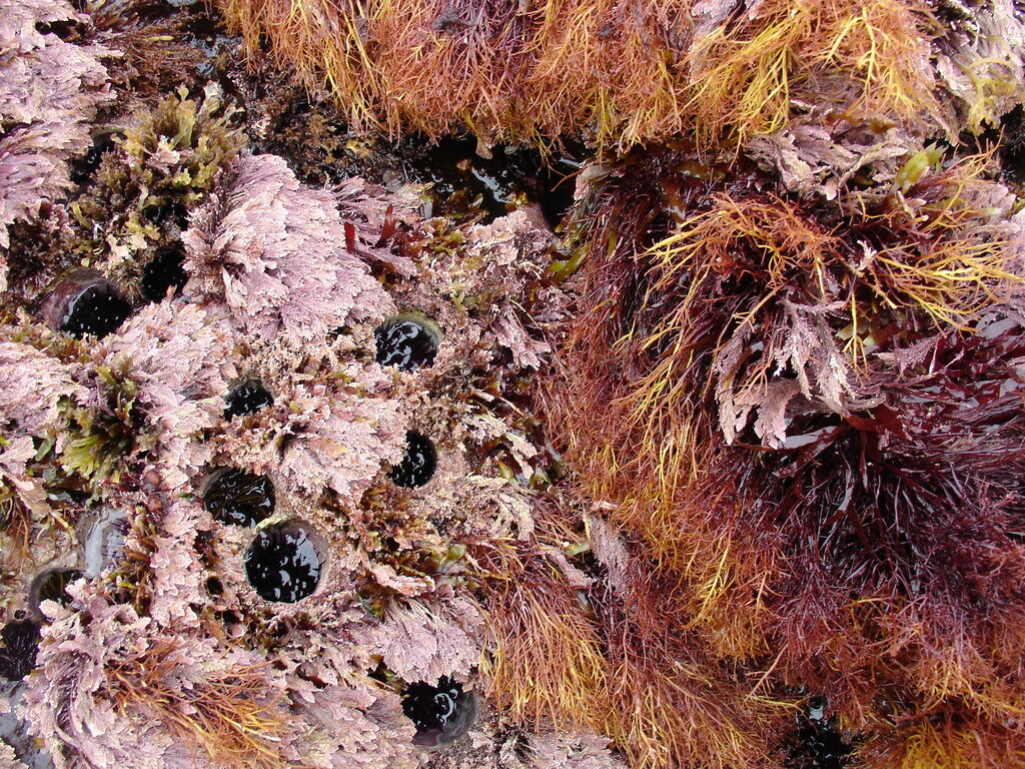
Patches of the agarophyte *Pterocladiella capillacea* and the calcareous *Ellisolandia elongata* at the low intertidal level (by the Island Aquatic Ecology Subgroup of cE3c-ABG).

Intertidal communities of São Miguel Island, as on the other islands of the Archipelago, are primarily dominated by macroalgae, which mainly exhibit a mosaic and/or zoned distribution pattern and have a predominance of algal turfs that cover the rocks as a carpet (Wallenstein et al. 2009), best seen when rocks are uncovered at low tide. There is a very distinct horizontal pattern of species distribution, with three major zones commonly found on bedrock and boulder shores (Neto 2000, Neto et al. 2005, Wallenstein et al. 2009b). The uppermost intertidal level is dominated by littorinids (Fig. 3), while the mid-level zone is usually characterised by a fringe of chthamalid barnacles (Fig. 4), in which sometimes algae and limpets can occur (Fig. 5), followed by a lower area, in which either algal turf (generally monospecific and usually composed of *Caulacanthus ustulatus* (Turner) Kützing) dominates (Fig. 6) or patches of the brown alga *Fucus spiralis* Linnaeus and the red agarophyte *Gelidium microdon* Kützing (Fig. 7) grow interspaced with barnacles and algal turf. The lowest intertidal zone, representing the transition to the sublittoral envrironment, is either dominated by algal turf (generally multispecific and commonly dominated by coralline algae, Fig. 8) or by various species of frondose algae growing in bands (e.g. the brown alga *Gongolaria abies-marina* (S.G.Gmelin) Kuntze, Fig. 9) or forming patches amongst and over turf species (e.g. the agarophyte *Pterocladiella capillacea* (S.G.Gmelin) Santelices & Hommersand and the calcareous *Ellisolandia elongata* (J.Ellis & Solander) K.R.Hind & G.W.Saunders, Fig. 10). The brown alga *Colpomenia sinuosa* (Mertens ex Roth) Derbès & Solier is very common at this level, growing epiphytically on several other algae. Seasonally, the red algae *Porphyra*/ *Neopyropia* and/or *Nemalion elminthoides* (Velley) Batters can be seen growing in patches at the mid-intertidal level. In some locations, the brown crust *Nemoderma tingitanum* Schousboe ex Bornet can be common at this shore level (Neto et al. 2005, Wallenstein et al. 2009b).

In spring and summer, considerable amounts of the introduced red alga *Asparagopsis armata* Harvey can be seen at the lower intertidal level, normally as an epiphyte on other algae (Neto, personal observation).

At cobble locations, the zonation pattern of macroalgae species is not clear (Costa 1994). The many microhabitats and substrate instability tend to mask and attenuate the limits of the biological zones. Nevertheless, in locations where cobbles are large and their size enlarges towards the sea (e.g. Fenais da Luz, north shore), the profile is steeper and usually the mid-intertidal level is dominated by the green macroalgae *Ulva linza* Linnaeus, *U. clathrata* (Roth) C.Agardh and *U. rigida* C.Agardh; the lower level is characterised by the presence of algal turf, mainly composed of *Jania crassa* J. V. Lamouroux and *Corallina officinalis* Linnaeus, with epiphytic Rhodophyta, such as *Asparagopsis armata* Harvey, phase *Falkenbergia rufolanosa* (Harvey) F.Schmitz, *Centroceras clavulatum* (C.Agardh) Montagne, *Ceramium ciliatum* (J.Ellis) Ducluzeau, *C. deslongchampsii* Chauvin ex Duby and *Polysiphonia atlantica* Kapraun & J.N.Norris. In locations with small cobbles (e.g. Caloura, south coast) or where there is a mixture of large and small cobbles (e.g. Povoação, south coast), the mid-intertidal level is usually characterised by fast growing algae, such as Cyanobacteria and the green algae *Ulva* spp., whereas the lowest level is also dominated by algal turf, but here mainly composed of *C. officinalis* and *C. clavulatum* (Caloura) or by *C. clavulatum*, *Chondracanthus acicularis* (Roth) Fredericq, *Jania* sp. and *Lophosiphonia* sp. (Povoação).

**Figure 11.**
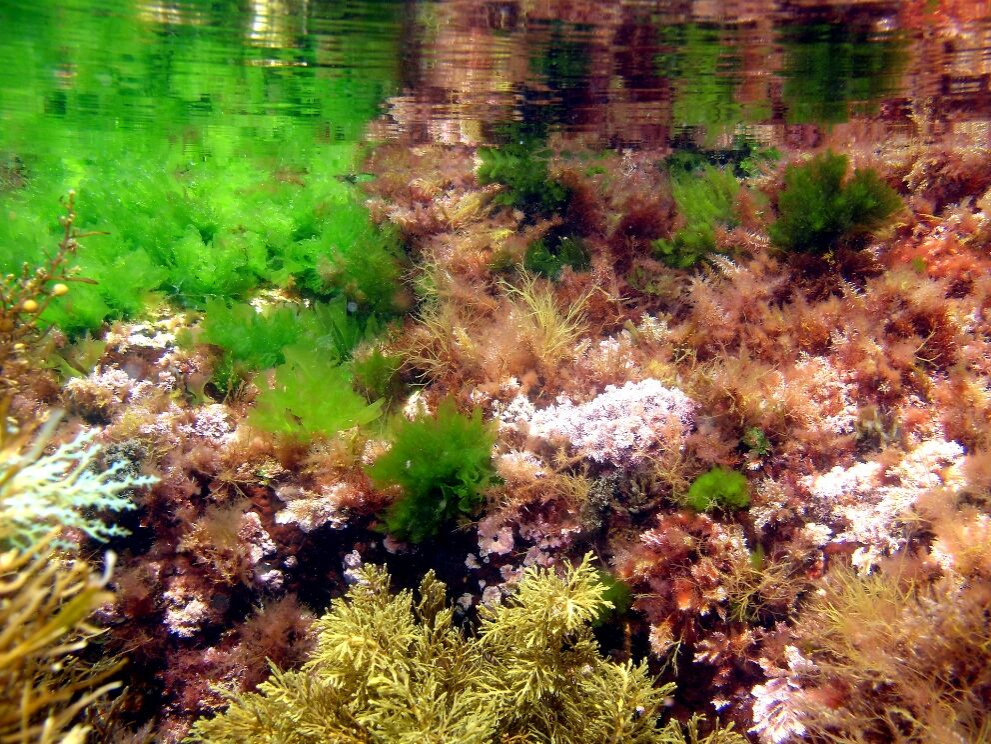
Low shore pool (by the Island Aquatic Ecology Subgroup of cE3c-ABG).

Important habitats at the shore level in bedrock locations are rock pools (Fig. 11). Differing in shape and size, they recreate a shallow subtidal habitat which may contain a rich diversity of marine algae and other marine organisms (Neto et al. 2005, Wallenstein et al. 2010).

The macroalgae diversity varies according to the pool location on the shore. Pools in the upper shore region are dominated by green algae, whilst those lower on the shore are dominated by red and brown algae. Similarly, faunal diversity in rock pools is greater at lower intertidal levels.

**Figure 12.**
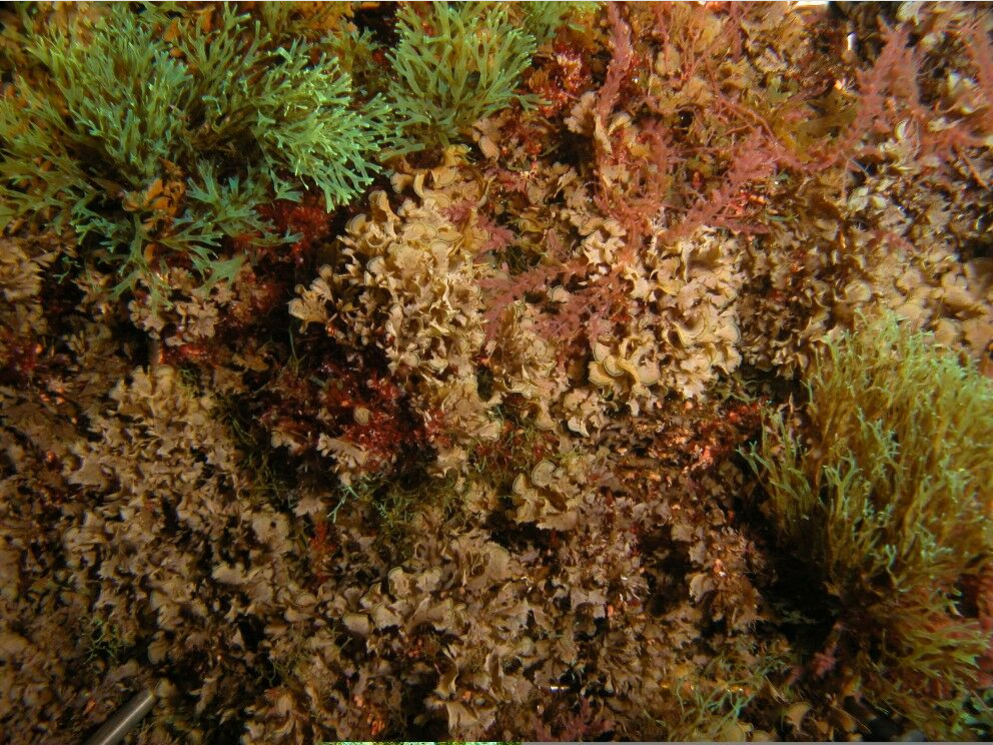
The frondose brown algae *Zonaria tournefortii* and *Dictyota* spp. at the deepest level sampled (by the Island Aquatic Ecology Subgroup of cE3c-ABG).

**Figure 13.**
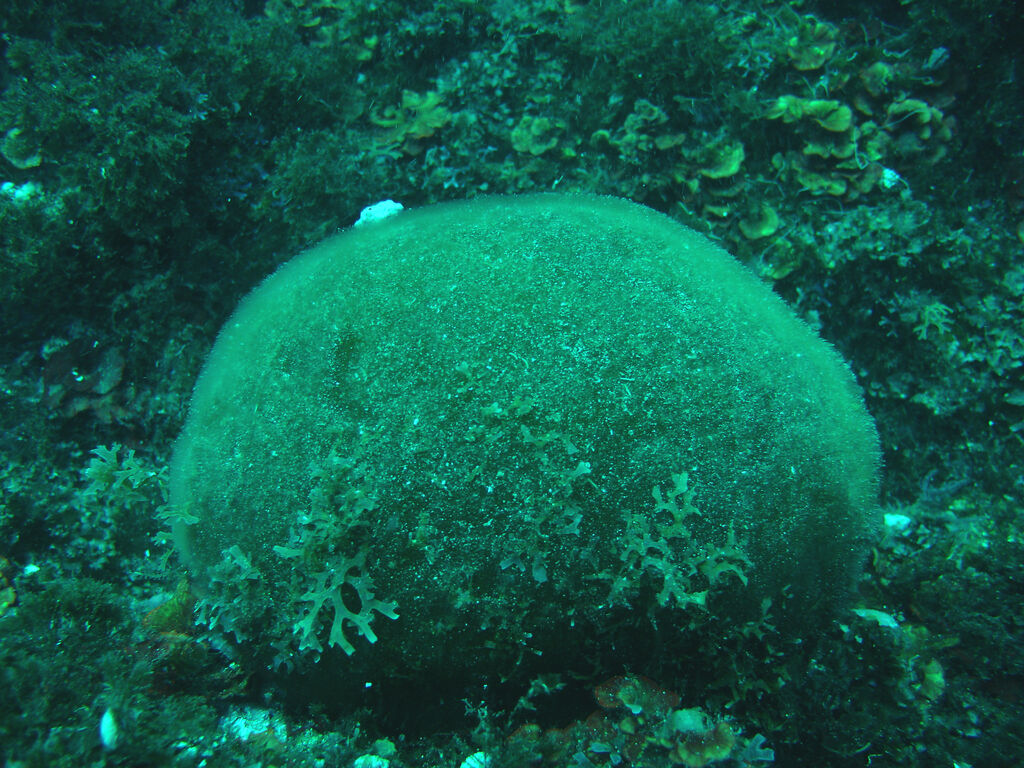
The Macaronesian endemic *Codium elisabethiae* on the shallow bottoms of São Miguel Island (by the Island Aquatic Ecology Subgroup of cE3c-ABG).

The adjacent submerged zone is also dominated by algal vegetation, with the rocky bottoms covered by more frondose macrophytes (Neto 2001, Wallenstein et al. 2009b), such as the red algae *Asparagopsis taxiformis* (Delile) Trevisan, *Ellisolandia elongata*, *Jania* spp., *Plocamium cartilagineum* (Linnaeus) P.S.Dixon, *Pterocladiella capillacea* and *Sphaerococcus coronopifolius* Stackhouse and the brown algae *Dictyopteris polypodioides* (A.P.De Candolle) J.V.Lamouroux, *Dictyota* spp., *Gongolaria abies marina* (S.G.Gmelin) Kuntze, *Halopteris scoparia* (Linnaeus) Sauvageau and *Zonaria tournefortii* (J.V.Lamouroux) Montagne (Fig. 12). The introduced red alga, *Symphyocladia marchantioides* (Harvey) Falkenberg, can be locally abundant below 15 m depth, usually as an ephiphyte on calcareous crusts; *Hypnea musciformis* (Wulfen) J.V.Lamouroux and *Dasya* spp. are other red algal species that can be locally abundant. The green species *Codium elisabethiae* (Fig. 13) and the brown species *Padina pavonica* (Linnaeus) Thivy can also be locally common, mainly in locations with sand influence (Neto 2001).

### Design description

The sampling referred to in this study was performed across littoral and sublittoral levels down to approximately 40 m. Each sampling location was visited several times and, on each occasion, a careful and extensive survey was undertaken to provide a good coverage of the area. Both presence recording and physical collections were made by walking over the intertidal shores during low tides or by SCUBA diving in the subtidal. The specimens collected were taken to the laboratory for identification and preservation and the resulting vouchers were deposited at the AZB Herbarium Ruy Telles Palhinha and the LSM - Molecular Systematics Laboratory at the Faculty of Sciences and Technology of the University of the Azores.

### Funding

This study was mainly financially supported by the following projects/scientific expeditions:

- Projects:
  - ABLA/MAC – “Associações Biológicas do Litoral Açoreano/Moluscos, Algas e Crustáceos”, funded by the Portuguese Science and Technology Foundation (1987-1991);
  - Azorean Algal Flora – “Studies on algal communities of São Miguel, Azores”, partially funded by CIRN/DB/UAc (1992-1996);
  - BIA - “Biodiversity of Azores Archipelago”, funded by the Portuguese Science and Technology Foundation. PRAXIS/2/2.1/BIA/169/94 (1996-1999);
  - BIOTOPE – “ Classification, mapping and modelling of Azorean littoral biotopes”, funded by the Portuguese Science and Technology Foundation, POCTI MGS/45319/2002 (2003-2006);
  - CAMAG/ORI – “Characterization of coastal water bodies on the islands of Santa Maria and São Miguel”, funded by the Regional Government of the Azores, Regional Secretariat for the Environment and the Sea, Regional Directorate for Planning and Water Resources (2008-2012);
  - GESMAR – “ Sustainable management of marine Resources”, funded by the EU Funding Programme III B 2000-2006, Açores-Madeira-Canárias, GESMAR/MAC/2/C068 (2009-2012);
  - PATELGENE – “ Genetic Structure of Azorean Limpets: Implications for Conservation and Marine Protected Areas”, funded by the Portuguese Science and Technology Foundation, PTDC/BIA-BIC/115837/2009 (2011-2014);
  - MACROBIOMOL – “Macroalgae biodiversity under a molecular view - for a better understanding of North Atlantic Biogeography”, funded by PTDC/MAR/114613/2009 (2011-2015);
  - ASMAS – “Açores: Stop-over for Marine Alien Species?”, funded by the Government of the Azores - Regional Secretariat for the Sea, Science and Technology, M2.1.2/I/032/2011 (2012-2016);
  - BUS – “ Urban Structures: a driver of biodiversity change in coastal ecosystems?”, funded by the Portuguese Science and Technology Foundation, PTDC/MAR-EST/2160/2012 (2013-2015);
  - ECOSUBVEG – “ Changes in submersed vegetation: assessing loss in ecosystems services from frondose to depauperate systems dominated by opportunistic vegetation”, funded by the Voluntary Scheme for Biodiversity and Ecosystem Services in Territories of the EU Outermost Regions and Oversees Countries and Territories, BEST 07.032700/2012/635752/SUB/B2 (2013-2016);
  - LAUMACAT - “ Diversity and phylogenetic relationships on the benthic marine algae with pharmacological potential: the *Laurencia* complex (Rhodophyta) in Macaronesian archipelagos, tropical and subtropical Atlantic”, funded by the Ministerio de Ciencia e Innovación, Dirección General de Investigación y Gestión del Plan Nacional de R+D+i, Subdirección General de Proyectos de Investigación, Gobierno de España (2010-2013) and by the São Paulo State Research Support Foundation (FAPESP), Brazil, Proc. 2014 / 00012-1 (2013 a 2016);
  - BALA – “Elaboration of the implementation program of the marine strategy framework directive - biodiversity of the coastal environments of the Azores” (2 /DRAM /2015), funded by the Government of the Azores - Regional Secretariat for the Sea, Science and Technology, Regional Directorate for Sea Affairs, GRA /SRMCT-DRAM, (2015);
  - PIMA – “Elaboration of the implementation program of the Marine Strategy Framework Directive - Marine Invasion Program in the Azores” (3/DRAM /2015), funded by the Government of the Azores - Regional Secretariat for the Sea, Science and Technology, Regional Directorate for Sea Affairs, GRA /SRMCT-DRAM, (2015);
  - ASPAZOR – “Ecosystem impacts and socioeconomic benefits of *Asparagopsis armata* in the Azores”, funded by the Regional Direction for Science, Technology. ACORES-01 -0145-FEDER-000060 (2016-2020);
  - PORBIOTA - “ACORES-01-0145-FEDER-000072 - AZORES BIOPORTAL”, funded by the Operational Programme Azores 2020 (85% ERDF and 15% regional funds) (2019-2021);
- Scientific Expeditions and campaigns:
  - “Campaign Macaronesia 2000”, under the project Macaronesia 2000 (2000-2001);
  - “Waitt Foundation”, under the projects BALA and PIMA (2016);
  - “BALA/PIMA”, under the projects BALA and PIMA (2018);
  - “PORBIOTA/2019” under the project ACORES-01-0145-FEDER-000072 - AZORES BIOPORTAL – PORBIOTA (2019);
- Other funds:
  - Portuguese National Funds, through FCT – the Portuguese Science and Technology Foundation, within the projects UID/BIA/00329/2013, 2015-2019, UID/BIA/00329/2020-2023 and UID/BIA/50027/2019, UID/BIA/50027/2013-2020, UID/Multi/04423/2013, PEst-C/MAR/LA0015/2013 and POCI-01-0145-FEDER-006821;
  - European Regional Development (ERD) funds through the Operational Programme for Competitiveness Factors (COMPETE);
  - Portuguese Regional Funds, through DRCT - Regional Directorate for Science and Technology, within several projects, 2019 and 2020 and SRMCT /DRAM - Regional Secretariat for the Sea, Science and Technology, Regional Directorate for Sea Affairs;
  - CIRN/DB/UAc (Research Centre for Natural Resources, Universidade dos Açores, Departamento de Biologia);
  - CIIMAR (Interdisciplinary Centre of Marine and Environmental Research, Porto, Portugal).

## Sampling methods

### Study extent

The present publication includes sampling performed over a relatively large area, covering littoral and sublittoral levels down to approximately 40 m around the Island (Table 1, Fig. 2).

### Sampling description

**Figure 14.**
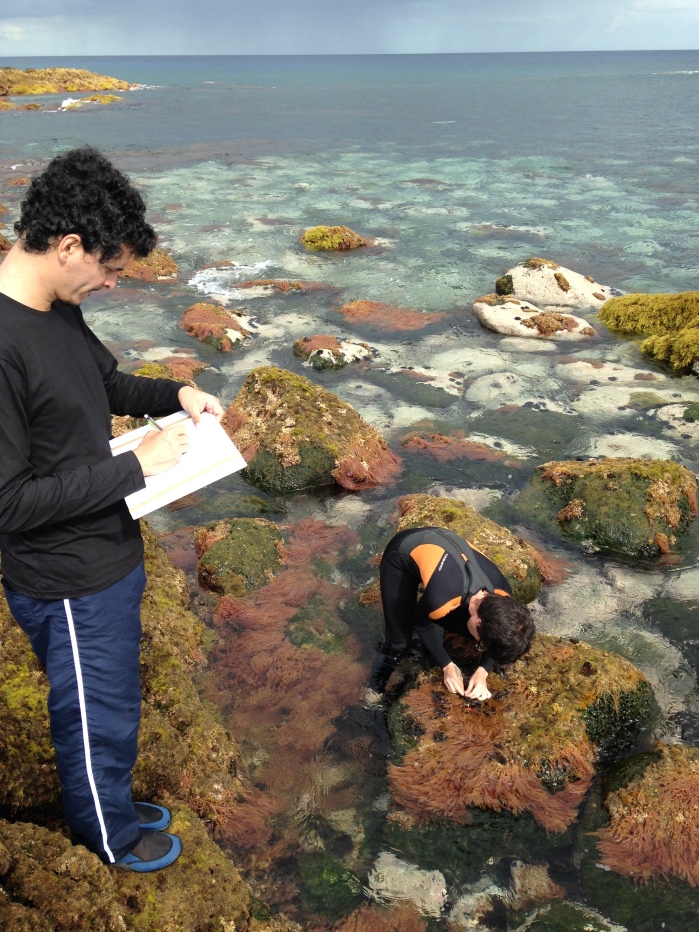
Quantitative recording of the presence and coverage of macroalgal species at the intertidal rocky habitat (by the Island Aquatic Ecology Subgroup of cE3c-ABG).

**Figure 15.**
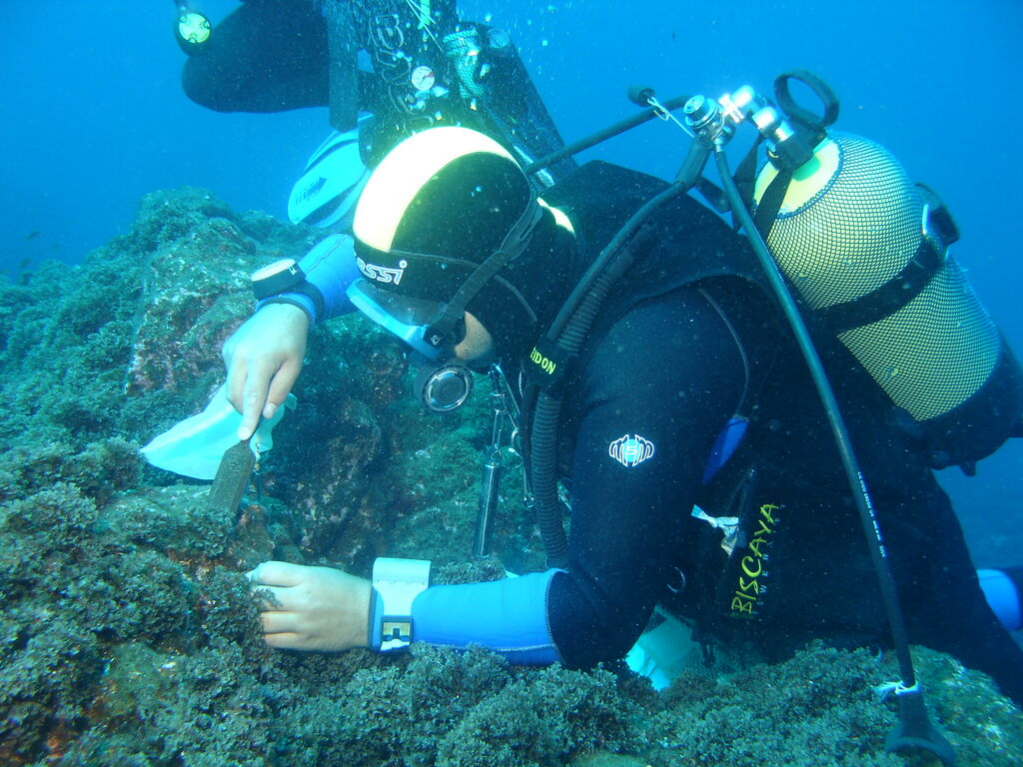
Collecting macroalgae in the subtidal of São Miguel Island (by the Island Aquatic Ecology Subgroup of cE3c-ABG).

Sampling involved species presence recording and/or specimen collecting at each sampling location. Species recording data were gathered by registering all species present in the sampled locations (Fig. 14). Destructive samples were obtained by scraping and/or manually collecting one or two specimens of every species found (Fig. 15). Intertidal collections were made during low tide by walking over the shores. Subtidal collections were made by SCUBA diving.

### Quality control

Each specimen collected was identified by trained taxonomists and involved morphological and anatomical observations of whole specimens by eye and/or of histological preparations under the microscope to determine the main diagnostic features of each species as described in literature.

### Step description

At the laboratory, specimen sorting and macroalgae identification followed standard procedures. A combination of morphological and anatomical characters and reproductive structures was used for species identification. For small and simple thalli, this required the observation of the entire thallus with the naked eye and/or using dissecting and compound microscopes. For larger and more complex algae, investigation of the thallus anatomy required histological preparations (longitudinal and transverse sections) or squashed preparations of mucilaginous thalli, sometimes after staining, to observe vegetative and reproductive structures and other diagnostic features.

The Azorean algal flora has components from several geographical regions, which implies difficulties in species identification. Floras and keys for the North Atlantic, Tropical Atlantic and Western Mediterranean were, therefore, used (e.g. Schmidt 1931, Taylor 1967, Taylor 1978, Levring 1974, Dixon and Irvine 1977, Lawson and John 1982, Irvine 1983, Gayral and Cosson 1986, Fletcher 1987, Afonso-Carrillo and Sansón 1989, Burrows 1991, Boudouresque et al. 1992, Cabioc'h et al. 1992, Maggs and Hommersand 1993, Irvine and Chamberlain 1994, Brodie et al. 2007, Lloréns et al. 2012, Rodríguez-Prieto et al. 2013). For more critical and taxonomically-difficult taxa, specimens were taken to the Natural History Museum (London) for comparison with collections there.

A reference collection was made for all collected specimens by assigning them a herbarium code number and depositing them at the AZB Herbarium Ruy Telles Palhinha and the LSM - Molecular Systematics Laboratory, University of Azores. Depending on the species and on planned further research, different methods of preservation were used, namely (i) wet collections using 5% buffered formaldehyde seawater and then replacing it by the fixing agent Kew (Bridsen and Forman 1999); (ii) dried collections, either by pressing the algae (most species) as described by Gayral and Cosson (1986) or by letting them air dry (calcareous species); and (iii) silica gel collections for molecular studies.

Nomenclatural and taxonomic status used here follow *Algaebase* (Guiry and Guiry 2021). The database was organised on FileMaker Pro.

## Geographic coverage

### Description

**São Miguel Island Description**: Azores, Portugal (approximately 37°54'58''N, 25°51'52''W).

### Coordinates

37°42'45''N and 37°54'57''N Latitude; 25°52'10''W and 25°08'06''W Longitude.

## Taxonomic coverage

### Description

All macroalgae were identified to genus or species level. In total, 431 taxa were identified belonging to 36 orders and 83 families, distributed amongst the phyla Rhodophyta (20 orders and 50 families), Chlorophyta (5 orders and 14 families) and Ochrophyta (11 orders and 19 families).

### Taxa included

**Table:** 

Rank	Scientific Name	Common Name
phylum	Rhodophyta	Red algae
phylum	Chlorophyta	Green algae
phylum	Ochrophyta	Brown algae

## Temporal coverage

### Notes

The sampling was performed on several occasions in the period between 1989 and 2019.

## Collection data

### Collection name

AZB | Marine macroalgae collection of São Miguel Island (Azores)-Campaign Macaronesia 2000; AZB | Marine macroalgae collection of São Miguel Island (Azores)-Occasional sampling; AZB | Marine macroalgae collection of São Miguel Island (Azores)-Project ABLA/MAC; AZB | Marine macroalgae collection of São Miguel Island (Azores)-Project ASPAZOR; AZB | Marine macroalgae collection of São Miguel Island (Azores)-Project Azorean Algal Flora; AZB | Marine macroalgae collection of São Miguel Island (Azores)-Project BIA (Biodiversity of Azores Archipelago); AZB | Marine macroalgae collection of São Miguel Island (Azores)-Project BIOTOPE; AZB | Marine macroalgae collection of São Miguel Island (Azores)-Project BUS; AZB | Marine macroalgae collection of São Miguel Island (Azores)-Project ECOSUBVEG; AZB | Marine macroalgae collection of São Miguel Island (Azores)-Project GESMAR; AZB | Marine macroalgae collection of São Miguel Island (Azores)-Project LAUMACAT; AZB | Marine macroalgae collection of São Miguel Island (Azores)-Project PATELGENE; LSM | Marine macroalgae collection of São Miguel Island (Azores)-Nordeste Expedition; LSM | Marine macroalgae collection of São Miguel Island (Azores)-Occasional sampling; LSM | Marine macroalgae collection of São Miguel Island (Azores)-Postdoc Manuela I Parente; LSM | Marine macroalgae collection of São Miguel Island (Azores)-Project LusoMarBol; LSM | Marine macroalgae collection of São Miguel Island (Azores)-Sabrina Expedition; LSM | Marine macroalgae collection of São Miguel Islands (Azores)-Master Project Artur Oliveira; LSM | Marine macroalgae collection of São Miguel Island (Azores)-Occasional sampling; LSM | Marine macroalgae collection of São Miguel Island (Azores)-Project MACROBIOMOL; AZB | Marine macroalgae occurrence of São Miguel Island (Azores)-Project BIA (Biodiversity of Azores Archipelago); AZB | Marine macroalgae occurrence of São Miguel Island (Azores)-Project BIOTOPE; AZB | Marine macroalgae occurrence of São Miguel Island (Azores)-Project BUS; AZB | Marine macroalgae occurrence of São Miguel Island (Azores)-Project GESMAR; AZB | Marine macroalgae occurrence of São Miguel Island (Azores)-Occasional sampling; AZB | Marine macroalgae occurrence of São Miguel Island (Azores)-Project Azorean Algal Flora; AZB | Marine macroalgae occurrence of São Miguel Island (Azores)-Project CAMAG-ORI-SMG; LSM | Marine macroalgae occurrence of São Miguel Island (Azores)-Occasional sampling; LSM | Marine macroalgae occurrence of São Miguel Island (Azores)-Campaign Waitt Foundation/2016; LSM | Marine macroalgae occurrence of São Miguel Island (Azores)-Campaign Waitt Foundation-BALA/PIMA/2016; LSM | Marine macroalgae occurrence of São Miguel Island (Azores)-PhD Andrea Z Botelho; LSM | Marine macroalgae occurrence of São Miguel Island (Azores)-PIMA/2016; LSM | Marine macroalgae occurrence of São Miguel Island (Azores)-PIMA/BALA/2019

### Collection identifier

3cee8546-66d5-49c1-b63f-8efd4227ccc9; 5f198d55-a6ad-42f8-9342-3d96513fe808; 15c76196-2b68-40cf-b49a-9392237f8d4d; c063f27f-5530-4e08-b50a-03843d61fb77; 996ba65a-d07a-4f4f-a17f-511a40983710; 445ca45c-3ba5-4a95-9289-39b944d6894e; 90fb970e-9be1-4caf-89ea-d3823235908a; 21215ae4-0e1f-44f0-95a8-dbed8022cfb0; 3dee61eb-0b56-4794-9d4e-75f2dce26918; 2840e5e1-4353-40f1-81c7-9c29ade05d2c; 7e12ab6e-a568-48aa-8ec3-0697ca734dac; ee3acc7e-abcf-4dec-9c08-961e15d4c029; c4d4cb43-19d4-4633-b252-0e73e6600800; 1fbe2045-3ccb-4e38-9058-d7927e76db62; 22941d45-0678-49fb-bdfe-8b0052ceb298; ef2b1875-8520-4520-b6e0-48a1039f9b1f; 64a1cad8-6242-4075-b762-64b84350864c; 4a97a5db-4970-4f54-b598-d1125a6b8c63; 494c9846-f867-4203-b476-42eb0789ca39; 54b8c165-ab17-4489-9382-e19f7a6af090; 3353c5f1-a12d-4c21-a0a2-c6c080d5d9fc; 2d7e1c20-23be-4e81-b349-4dba060ef8de; 5b048916-da07-4366-97f6-31741d804e51; 7a3f16c8-fb0e-4118-bf69-54dd20024146; 15799ce3-106e-48ff-933d-3d6a0a5b079d; bd4dddc7-c708-4b0d-b912-e9a0a14a87f8; f9a307a6-1137-4265-baae-85924ef72ae7; b252cbd3-385a-4808-bd39-05add8d8eca0; 5efb9cd5-d89c-4ee9-b29d-9c4ce401e186; fd958ad3-143a-4be7-bc89-0b58879c36ef; 52e0ab57-3fe9-436e-90d8-5ea2e1f4899e; 57fc6968-c1f3-4802-9c73-0609a61b8b10; f5b2f2cc-58c8-4bdf-9ab3-1fe3e5feea65

### Parent collection identifier

AZB Herbarium Ruy Telles Palhinha, Faculty of Sciences and Technology of the University of the Azores; AZB Herbarium Ruy Telles Palhinha, Faculty of Sciences and Technology of the University of the Azores; AZB Herbarium Ruy Telles Palhinha, Faculty of Sciences and Technology of the University of the Azores; AZB Herbarium Ruy Telles Palhinha, Faculty of Sciences and Technology of the University of the Azores; AZB Herbarium Ruy Telles Palhinha, Faculty of Sciences and Technology of the University of the Azores; AZB Herbarium Ruy Telles Palhinha, Faculty of Sciences and Technology of the University of the Azores; AZB Herbarium Ruy Telles Palhinha, Faculty of Sciences and Technology of the University of the Azores; AZB Herbarium Ruy Telles Palhinha, Faculty of Sciences and Technology of the University of the Azores; AZB Herbarium Ruy Telles Palhinha, Faculty of Sciences and Technology of the University of the Azores; AZB Herbarium Ruy Telles Palhinha, Faculty of Sciences and Technology of the University of the Azores; AZB Herbarium Ruy Telles Palhinha, Faculty of Sciences and Technology of the University of the Azores; AZB Herbarium Ruy Telles Palhinha, Faculty of Sciences and Technology of the University of the Azores; LSM - Molecular Systematics Laboratory, Faculty of Sciences and Technology of the University of the Azores; LSM - Molecular Systematics Laboratory, Faculty of Sciences and Technology of the University of the Azores; LSM - Molecular Systematics Laboratory, Faculty of Sciences and Technology of the University of the Azores; LSM - Molecular Systematics Laboratory, Faculty of Sciences and Technology of the University of the Azores; LSM - Molecular Systematics Laboratory, Faculty of Sciences and Technology of the University of the Azores; LSM - Molecular Systematics Laboratory, Faculty of Sciences and Technology of the University of the Azores; LSM - Molecular Systematics Laboratory, Faculty of Sciences and Technology of the University of the Azores; LSM - Molecular Systematics Laboratory, Faculty of Sciences and Technology of the University of the Azores; Not applicable; Not applicable; Not applicable; Not applicable; Not applicable; Not applicable; Not applicable; Not applicable; Not applicable; Not applicable; Not applicable; Not applicable; Not applicable

### Specimen preservation method

Air dry, Dried and pressed; Wet (Formalin; fixing agent Kew), Silica gel

### Curatorial unit

AZB Herbarium Ruy Telles Palhinha, Faculty of Sciences and Technology of the University of the Azores

## Usage licence

### Usage licence

Creative Commons Public Domain Waiver (CC-Zero)

## Data resources

### Data package title

Marine algal flora of São Miguel Island, Azores

### Resource link

http://ipt.gbif.pt/ipt/resource?r=sao_miguel_macroalgal_flora

### Alternative identifiers

https://www.gbif.org/dataset/322b5629-997c-4986-ada9-7d9d078d8648;  https://doi.org/10.15468/xtuzd3

### Number of data sets

1

### Data set 1.

#### Data set name

Marine algal flora of São Miguel Island, Azores

#### Data format

Darwin Core Archive

#### Number of columns

50

#### Download URL

http://ipt.gbif.pt/ipt/resource?r=sao_miguel_macroalgal_flora&amp;v=1.0

#### Data format version

1.3

#### Description

This data paper presents physical and occurrence data from macroalgal surveys undertaken on São Miguel Island between 1989 and 2019 (Neto et al. 2021b). The dataset submitted to GBIF is structured as a sample event dataset, with two tables: event (as core) and occurrences. The data in this sampling event resource have been published as a Darwin Core Archive (DwCA), which is a standardised format for sharing biodiversity data as a set of one or more data tables. The core data table contains 506 records (eventID). The extension data table has 12,781 occurrences. An extension record supplies extra information about a core record. The number of records in each extension data table is illustrated in the IPT link. This IPT archives the data and thus serves as the data repository. The data and resource metadata are available for downloading in the downloads section.

**Data set 1.** 

Column label	Column description
eventID	Identifier of the event, unique for the dataset
country	Country of the sampling site
countryCode	Code of the country where the event occurred
stateProvince	Name of the region
island	Name of the island
municipality	Name of the municipality
locality	Name of the locality
locationID	Identifier of the location
decimalLatitude	The geographic latitude of the sampling site
decimalLongitude	The geographic longitude of the sampling site
geodeticDatum	The spatial reference system upon which the geographic coordinates are based
coordinateUncertaintyInMetres	The horizontal distance (in metres) from the given decimalLatitude and decimalLongitude describing the smallest circle containing the whole of the Location
eventDate	Time interval when the event occurred
year	The year of the event
samplingProtocol	Sampling method used during an event
locationRemarks	Zonation level
minimumDepthInMetres	The minimum depth in metres where the specimen was found
maximumDepthInMetres	The maximum depth in metres where the specimen was found
eventRemarks	Notes about the event
occurrenceID	Identifier of the record, coded as a global unique identifier
institutionID	The identifier for the institution having custody of the object or information referred to in the record
institutionCode	The acronym of the institution having custody of the object or information referred to in the record
collectionID	An identifier of the collection to which the record belongs
collectionCode	The name of the collection from which the record was derived
datasetName	The name identifying the dataset from which the record was derived
kingdom	Kingdom name
phylum	Phylum name
class	Class name
order	Order name
family	Family name
genus	Genus name
specificEpithet	The name of the first or species epithet of the scientificName
infraspecificEpithet	The name of the lowest or terminal infraspecific epithet of the scientificName, excluding any rank designation
acceptedNameUsage	The specimen accepted name, with authorship
previousIdentifications	Previous name of the specimen, with authorship
scientificName	The name without authorship applied on the first identification of the specimen
scientificNameAuthorship	The authorship information for the scientificName formatted according to the conventions of the applicable nomenclaturalCode
taxonRank	The taxonomic rank of the most specific name in the scientificName
basisOfRecord	The specific nature of the data record
habitat	Description of the habitat where the specimen was found
organismQuantityType	The type of quantification system used to quantify the organisms
organismQuantity	Percentage of the organism coverage
recordedBy	Person(s) responsible for sampling
catalogNumber	Identifying code for a unique sample lot in a biological collection
identifiedBy	Person(s) responsible for taxa identification
type	The nature of the resource
preparations	The preservation method used for the specimen
establishmentMeans	The establishment status of the organism in the study region
occurrenceRemarks	New record status assignment
licence	Reference to the licence under which the record is published

## Additional information

This paper accommodates the 12,781 specimens of macroalgae recorded from São Miguel Island in 431 taxa comprising 323 confirmed species (Tables 2, 3) and 108 taxa identified only to genus level. The confirmed species (Table 3) include 212 Rhodophyta, 48 Chlorophyta and 63 Ochrophyta (Phaeophyceae). Of these, 61 species are newly recorded to the Island (42 Rhodophyta, 9 Chlorophyta and 10 Ochrophyta). Most species are native, Predaea feldmannii subsp. azorica is an Azorean endemic, whereas the rhodophyta *Botryocladia macaronesica*, *Laurencia viridis*, *Millerella tinerfensis*, *Phyllophora gelidioides* and the Chlorophyta *Codium elisabethiae* are Macaronesian endemics. Nineteen species represent introductions to the algal flora (the Rhodophyta *Antithamnion diminuatum* Wollaston, *Antithamnion hubbsii* E.Y.Dawson, *Antithamnionella spirographidis* (Schiffner) E.M.Wollaston, *Antithamnionella ternifolia* (J.D.Hooker & Harvey) Lyle, *Asparagopsis armata, Grallatoria reptans* M.Howe, *Gymnophycus hapsiphorus* Huisman & Kraft, *Laurencia brongniartii* J.Agardh, *Laurencia dendroidea* J.Agardh, *Neoizziella divaricata* (C.K.Tseng) S.-M.Lin, S.-Y.Yang & Huisman, *Scageliopsis patens* E.M.Wollaston, *Symphyocladia marchantioides*, *Xiphosiphonia pennata* (C.Agardh) Savoie & G.W.Saunders and *Xiphosiphonia pinnulata* (Kützing) Savoie & G.W.Saunders; the Chlorophyta *Caulerpa prolifera* (Forsskål) J.V.Lamouroux and Codium fragile subsp. fragile (Suringar) Hariot; and the Ochrophyta *Papenfussiella kuromo* (Yendo) Inagaki and *Petalonia binghamiae* (J.Agardh) K.L.Vinogradova. Thirty-two species have an uncertain status (21 Rhodophyta, 5 Chlorophyta and 6 Ochrophyta).

Many species were only sporadically recorded, but 10 were widely recorded around the Island and occurred quite abundantly in some locations, namely: the Rhodophyta *Acrosorium ciliolatum* (Harvey) Kylin, *Chondracanthus acicularis*, *Gelidium microdon* and *Pterocladiella capillacea*; and the Ochrophyta *Colpomenia sinuosa*, *Gongolaria abies-marina*, *Halopteris filicina* (Grateloup) Kützing, *Halopteris scoparia*, *Padina pavonica* and *Zonaria tournefortii*.

This paper increases the total of macroalgae species previously listed for São Miguel Island by 63 (44 Rhodophyta, 9 Chlorophyta and 10 Ochrophyta). When compared with the other Azorean Islands (Table 4), São Miguel has the highest number of species in all phylla, which reflects the greater amount of research undertaken on this Island on a more regular basis, involving both temporal and long-term studies.

In general and in keeping with other warm-water areas of the North Atlantic Ocean, the Azorean macroalgae flora has a larger proportion of red seaweeds.

A relatively high number of non-native species has been recorded on São Miguel Island (see Tables 2, 3), similarly to what has been reported for Santa Maria (Neto et al. 2020d), Terceira (Neto et al. 2020a), Graciosa (Neto et al. 2020c), Pico (Neto et al. 2020b) and Flores and Corvo (Neto et al. 2021a). Research over the past 15 years (Cardigos et al. 2006, Micael et al. 2014, Vaz-Pinto et al. 2014, Chainho et al. 2015, Cacabelos et al. 2019, Cacabelos et al. 2020, Martins et al. 2019, Parente et al. 2019) has indicated that the arrival of non-native species is increasing in the Azores, which is very likely to be related to the geostrategic position of the Archipelago as a “crossroad” in the distribution of marine algae in the North Atlantic, with documented exchanges with European, Mediterranean and American coasts. This may favour the arrival of new species via maritime traffic, both commercial and recreational (hull fouling, ballast waters), which may be a high risk to local marine ecosystems, as non-indigenous species can become invasive, resulting in impacts on ecosystem services and biodiversity (e.g. Katsanevakis et al. 2014).

The discovery of the new macroalgae records on São Miguel Island (present study) and on the other Azorean Islands (Neto et al. 2020a, Neto et al. 2020b, Neto et al. 2020c, Neto et al. 2020d, Neto et al. 2021a), demonstrates the need for continuing taxonomic and floristic studies in this region of the Atlantic Ocean. The biogeographically-variable nature of the new records found confirms the overall mixed nature of the marine algal flora of the Azores with elements shared with Macaronesia, the Mediterranean Sea, Atlantic Europe and the subtropical and tropical Atlantic America.

### Taxonomic mismatch

A mismatch regarding the GBIF backbone taxonomy of some of the macroalgae species names was identified as detailed in Suppl. material 1.

## Supplementary Material

F1432CB9-177B-53C6-96B4-A8A0DBE28B2A 10.3897/BDJ.9.e64969.suppl1 Supplementary material 1 DP-SMG-id_15785_normalized.csv Data type Macroalgae taxonomic mismatching Brief description GBIF does not have the more actualised nomenclature for some of the macroalgae species names. Therefore, the matching tools of its platform were applied to the species list, as required by Pensoft's data auditor, to identify the problematic taxonomic situations. The resulting file (DP-SMG-id_15785_normalized.csv) is included here, since the names will not be immediately updated in the GBIF Taxonomic Backbone. A request was already sent to GBIF helpdesk to solve this situation. File: oo_505943.csv https://binary.pensoft.net/file/505943 Ana I Neto

## Figures and Tables

**Table 1.** Information and location of the sampling sites on São Miguel Island

Location N0	Location ID	Municipality	Locality	Latitude / Longitude	Littoral zone
1	SMG_L_APs	Lagoa	Água de Pau | Subtidal	37°43'08''N, 25°27'45''W	Subtidal
2	SMG_L_APsE	Lagoa	Água de Pau | Subtidal E	37°43'10''N, 25°27'44''W	Subtidal
3	SMG_L_APsW	Lagoa	Água de Pau | Subtidal W	37°43'27''N, 25°27'16''W	Subtidal
4	SMG_L_Avb	Lagoa	Atalhada | Viteleiro | baía	37°44'38''N, 25°23'23''W	Subtidal
5	SMG_L_Avem	Lagoa	Atalhada | Viteleiro | Entre-marés	37°44'43''N, 25°23'24''W	Intertidal
6	SMG_L_Cbab	Lagoa	Caloura | Baixa da Areia | baía	37°42'50''N, 25°28'11''W	Subtidal
7	SMG_L_Ccb	Lagoa	Caloura | Cerco | baía	37°42'24''N, 25°29'30''W	Subtidal
8	SMG_L_CcbW	Lagoa	Caloura | Cerco | baía W	37°42'21''N, 25°29'07''W	Subtidal
9	SMG_L_Ccem	Lagoa	Caloura | Cerco | Entre-marés	37°42'27''N, 25°29'27''W	Intertidal
10	SMG_L_Cepgb	Lagoa	Caloura | Entre Porto e Galera | baía	37°42'24''N, 25°29'52''W	Subtidal
11	SMG_L_Cgb	Lagoa	Caloura | Galera | baía	37°42'10''N, 25°29'27''W	Subtidal
12	SMG_L_Chem	Lagoa	Caloura | Hotel | Entre-marés	37°42'50''N, 25°28'59''W	Intertidal
13	SMG_L_Cpb	Lagoa	Caloura | porto | baía	37°42'45''N, 25°30'15''W	Subtidal
14	SMG_L_Cpem	Lagoa	Caloura | porto | Entre-marés	37°42'47''N, 25°30'15''W	Intertidal
15	SMA_L_Crcb	Lagoa	Caloura | Ribeira Chã | baía	37°42'45''N, 25°30'16''W	Subtidal
16	SMG_L_Lbp	Lagoa	Lagoa | Bairro dos Pescadores	37°44'23''N, 25°25'00''W	Subtidal
17	SMG_L_Lcrb	Lagoa	Lagoa | Cruzeiro | baía	37°44'34''N, 25°25'56''W	Subtidal
18	SMG_L_Lcrem	Lagoa	Lagoa | Cruzeiro | Entre-marés	37°44'32''N, 25°25'49''W	Intertidal
19	SMG_L_Lovem	Lagoa	Lagoa | Observatório vulcanológico | Entre-marés	37°44'31''N, 25°24'44''W	Intertidal
20	SMG_L_Lpib	Lagoa	Lagoa | Piscina | baía	37°44'26''N, 25°25'33''W	Subtidal
21	SMG_L_Lpiem	Lagoa	Lagoa | Piscina | Entre-marés	37°44'29''N, 25°25'34''W	Intertidal
22	SMG_L_Lpb	Lagoa	Lagoa | porto | baía	37°44'26''N, 25°25'25''W	Subtidal
23	SMG_L_Pepem	Lagoa	Pisão | Entre praias | Entre-marés	37°43'02''N, 25°31'12''W	Intertidal
24	SMG_N_AN	Nordeste	Achada do Nordeste	37°51'34''N, 25°43'23''W	Intertidal
25	SMG_N_LGpsem	Nordeste	Lombo Gordo | Ponta do Sossego | Entre-marés	37°47'18''N, 25°51'27''W	Intertidal
26	SMG_N_Nb6	Nordeste	Nordeste | baía 6	37°50'21''N, 25°52'15''W	Subtidal
27	SMG_N_Nb7	Nordeste	Nordeste | baía 7	37°48'55''N, 25°53'22''W	Subtidal
28	SMG_N_Npinb	Nordeste	Nordeste | Piscinas Naturais | baía	37°50'39''N, 25°51'17''W	Subtidal
29	SMG_N_Npmb	Nordeste	Nordeste | Ponta da Madrugada | baía	37°49'30''N, 25°52'25''W	Subtidal
30	SMG_N_Npmqb	Nordeste	Nordeste | Ponta da Marquesa | baía	37°47'59''N, 25°51'35''W	Subtidal
31	SMG_N_Npaem	Nordeste	Nordeste | Ponta do Arnel | Entre-marés	37°49'21''N, 25°51'49''W	Intertidal
32	SMG_PD_Bab	Ponta Delgada	Bretanha | Ajuda | baía	37°54'02''N, 25°14'56''W	Subtidal
33	SMG_PD_Baem	Ponta Delgada	Bretanha | Ajuda | Entre-marés	37°54'00''N, 25°15'00''W	Intertidal
34	SMG_PD_Cb	Ponta Delgada	Candelária | baía	37°49'15''N, 25°10'04''W	Subtidal
35	SMG_PD_CPpb	Ponta Delgada	Capelas | porto | baía	37°50'32''N, 25°18'46''W	Subtidal
36	SMG_PD_FLem	Ponta Delgada	Fenais da Luz | Entre-marés	37°49'54''N, 25°22'23''W	Intertidal
37	SMG_PD_Fb	Ponta Delgada	Ferraria | baía	37°51'26''N, 25°08'51''W	Subtidal
38	SMG_PD_Fem	Ponta Delgada	Ferraria | Entre-marés	37°51'30''N, 25°08'50''W	Intertidal
39	SMG_PD_Ftb	Ponta Delgada	Feteiras | baía	37°46'57''N, 25°13'24''W	Subtidal
40	SMG_PD_Ftem	Ponta Delgada	Feteiras | Entre-marés	37°48'13''N, 25°11'47''W	Intertidal
41	SMG_PD_Ftpfgs	Ponta Delgada	Feteiras | Ponta da Fonte Grande | Subtidal	37°46'12''N, 25°13'48''W	Subtidal
42	SMG_PD_Ftpem	Ponta Delgada	Feteiras | Porto | Entre-marés	37°48'12''N, 25°11'49''W	Intertidal
43	SMG_PD_Mib	Ponta Delgada	Mosteiros | Ilhéus | baía	37°53'20''N, 25°10'00''W	Subtidal
44	SMG_PD_Mpinb	Ponta Delgada	Mosteiros | piscinas naturais | baía	37°54'02''N, 25°10'31''W	Subtidal
45	SMG_PD_Mpinemb	Ponta Delgada	Mosteiros | piscinas naturais | Entre-marés | Blocos	37°53'56''N, 25°10'58''W	Intertidal
46	SMG_PD_Mpinemc1	Ponta Delgada	Mosteiros | piscinas naturais | Entre-marés | Calhau 1	37°53'58''N, 25°10'45''W	Intertidal
47	SMG_PD_Mpinemc2	Ponta Delgada	Mosteiros | piscinas naturais | Entre-marés | Calhau 2	37°53'57''N, 25°10'56''W	Intertidal
48	SMG_PD_Mpineme1	Ponta Delgada	Mosteiros | piscinas naturais | Entre-marés | Escoada 1	37°54'01''N, 25°10'50''W	Intertidal
49	SMG_PD_Mpineme2	Ponta Delgada	Mosteiros | piscinas naturais | Entre-marés | Escoada 2	37°53'59''N, 25°10'44''W	Intertidal
50	SMG_PD_Mpineme3	Ponta Delgada	Mosteiros | piscinas naturais | Entre-marés | Escoada 3	37°53'59''N, 25°10'46''W	Intertidal
51	SMG_PD_Mpineme4	Ponta Delgada	Mosteiros | piscinas naturais | Entre-marés | Escoada 4	37°54'02''N, 25°10'54''W	Intertidal
52	SMG_PD_Mpinpo	Ponta Delgada	Mosteiros | piscinas naturais | Poças	37°54'01''N, 25°10'48''W	Intertidal
53	SMG_PD_Mpem	Ponta Delgada	Mosteiros | porto | Entre-marés	37°53'33''N, 25°10'42''W	Intertidal
54	SMG_PD_Mpremw	Ponta Delgada	Mosteiros | Praia | Entre-marés (W)	37°53'20''N, 25°10'27''W	Intertidal
55	SMG_PD_Pdcacb	Ponta Delgada	Ponta Delgada | Calheta | atrás da cadeia | baía	37°44'30''N, 25°20'46''W	Subtidal
56	SMG_PD_PDeb	Ponta Delgada	Ponta Delgada | Etar | baía	37°44'27''N, 25°21'02''W	Subtidal
57	SMG_PD_PDeem	Ponta Delgada	Ponta Delgada | Etar | Entre-marés	37°44'29''N, 25°21'02''W	Intertidal
58	SMG_PD_PDeem	Ponta Delgada	Ponta Delgada | Etar | Entre-marés	37°44'29''N, 25°21'02''W	Subtidal
59	SMG_PD_PDmle	Ponta Delgada	Ponta Delgada | marina | lado externo	37°44'22''N, 25°20'35''W	Subtidal
60	SMG_PD_Pdmleem	Ponta Delgada	Ponta Delgada | marina | lado externo | Entre-marés	37°44'22''N, 25°20'29''W	Intertidal
61	SMG_PD_PDmli	Ponta Delgada	Ponta Delgada | marina | lado interno	37°44'26''N, 25°20'29''W	Subtidal
62	SMG_PD_PDmn	Ponta Delgada	Ponta Delgada | marina nova	37°44'20''N, 25°20'05''W	Intertidal
63	SMG_PD_PDmn	Ponta Delgada	Ponta Delgada | marina nova	37°44'20''N, 25°20'05''W	Subtidal
64	SMG_PD_PDpe	Ponta Delgada	Ponta Delgada | pesqueiro	37°44'21''N, 25°20'18''W	Subtidal
65	SMG_PD_PDple	Ponta Delgada	Ponta Delgada | porto | lado externo	37°44'06''N, 25°20'28''W	Subtidal
66	SMG_PD_PDpli	Ponta Delgada	Ponta Delgada | porto | lado interno	37°44'11''N, 25°20'32''W	Subtidal
67	SMG_PD_PDscb	Ponta Delgada	Ponta Delgada | Santa Clara | baía	37°43'53''N, 25°18'59''W	Subtidal
68	SMG_PD_PDscfem	Ponta Delgada	Ponta Delgada | Santa Clara | Farol | Entre-marés	37°43'58''N, 25°18'46''W	Intertidal
69	SMG_PD_PDscpoem	Ponta Delgada	Ponta Delgada | Santa Clara | pontão | Entre-marés	37°43'58''N, 25°18'45''W	Intertidal
70	SMG_PD_Popmem	Ponta Delgada	Pópulo | Praia das milícias | Entre-marés	37°44'58''N, 25°22'38''W	Intertidal
71	SMG_PD_Poppem	Ponta Delgada	Pópulo | Praia pequena | Entre-marés	37°44'56''N, 25°22'59''W	Intertidal
72	SMG_PD_Rb	Ponta Delgada	Relva | baía	37°45'57''N, 25°15'21''W	Subtidal
73	SMG_R_bes	Ponta Delgada	Relva | Baixa do Espelho | Subtidal	37°45'41''N, 25°15'29''W	Subtidal
74	SMG_PD_SAACem	Ponta Delgada	Santo António Além Capelas | Entre-marés	37°51'52''N, 25°18'00''W	Intertidal
75	SMG_PD_SRd	Ponta Delgada	São Roque | Dori	37°44'42''N, 25°22'22''W	Subtidal
76	SMG_PD_SRfcem	Ponta Delgada	São Roque | Forno da Cal | Entre-marés	37°44'39''N, 25°21'34''W	Intertidal
77	SMG_PD_SRpb	Ponta Delgada	São Roque | Pranchinha | baía	37°44'37''N, 25°21'08''W	Subtidal
78	SMG_PD_SRpem	Ponta Delgada	São Roque | Pranchinha | Entre-marés	37°44'38''N, 25°21'07''W	Intertidal
79	SMG_PD_SRrcb	Ponta Delgada	São Roque | Rosto do Cão | baía	37°44'36''N, 25°21'39''W	Subtidal
80	SMG_PD_SRrcem1	Ponta Delgada	São Roque | Rosto do Cão | Entre-marés 1	37°44'40''N, 25°21'37''W	Intertidal
81	SMG_PD_SRrcem2	Ponta Delgada	São Roque | Rosto do Cão | Entre-marés 2	37°44'38''N, 25°21'40''W	Intertidal
82	SMG_PD_SRrcem3	Ponta Delgada	São Roque | Rosto do Cão | Entre-marés 3	37°44'40''N, 25°21'39''W	Intertidal
83	SMG_PD_SVpobe	Ponta Delgada	São Vicente | Poços | baía (E)	37°50'06''N, 25°20'04''W	Subtidal
84	SMG_PD_SVpobw	Ponta Delgada	São Vicente | Poços | baía (W)	37°50'04''N, 25°19'55''W	Subtidal
85	SMG_PD_SVpoeme	Ponta Delgada	São Vicente | Poços | Entre-marés (E)	37°50'02''N, 25°19'53''W	Intertidal
86	SMG_PD_SVpoemw	Ponta Delgada	São Vicente | Poços | Entre-marés (W)	37°50'03''N, 25°19'53''W	Intertidal
87	SMG_PD_SVpoi	Ponta Delgada	São Vicente | Poços | ilhéu	37°50'03''N, 25°20'02''W	Subtidal
88	SMG_P_bls	Povoação	Baixa da Lobeira | Subtidal	37°43'14''N, 25°40'52''W	Subtidal
89	SMG_P_brqs	Povoação	Baixa da Ribeira Quente | Subtidal	37°43'26''N, 25°41'22''W	subtidal
90	SMG_P_FTem	Povoação	Faial da Terra | Entre-marés	37°44'20''N, 25°48'12''W	Intertidal
91	SMG_P_RQbf	Povoação	Ribeira Quente | baía | Fumarolas	37°43'36''N, 25°41'22''W	Subtidal
92	SMG_P_RQbr	Povoação	Ribeira Quente | Baixa da Ribeira	37°43'59''N, 25°42'16''W	Subtidal
93	SMG_P_RQborb	Povoação	Ribeira Quente | Boca da Ribeira | baía	37°43'59''N, 25°42'23''W	Subtidal
94	SMG_P_RQrcem	Povoação	Ribeira Quente | Rua do Castelo | Entre-marés	37°43'43''N, 25°41'32''W	Intertidal
95	SMG_RG_apgrpcpm21s	Ribeira Grande	Área Protegida de Gestão de Recursos da Ponta do Cintrão – Ponta da Maia (SMG21) | Subtidal	37°50'34''N, 25°30'51''W	Subtidal
96	SMG_RG_FAem	Ribeira Grande	Fenais da Ajuda | Entre-marés	37°51'56''N, 25°41'01''W	Intertidal
97	SMG_RG_Mamem	Ribeira Grande	Maia | Alameda do Mar | Entre-marés	37°50'03''N, 25°36'17''W	Intertidal
98	SMG_RG_M_cms	Ribeira Grande	Maia | Cabecinhos da Maia | Subtidal	37°50'44''N, 25°36'00''W	Subtidal
99	SMG_RG_Mfmb	Ribeira Grande	Maia | Frade da Maia | baía	37°50'17''N, 25°36'07''W	Subtidal
100	SMG_RG_Mlmb	Ribeira Grande	Maia | Lomba da Maia | baía	37°50'56''N, 25°38'43''W	Subtidal
101	SMG_RG_Mpinpb	Ribeira Grande	Maia | Piscinas naturais | pontas | baía	37°50'08''N, 25°36'20''W	Subtidal
102	SMG_RG_Mpinpem	Ribeira Grande	Maia | Piscinas naturais | pontas | Entre-marés	37°50'10''N, 25°36'23''W	Intertidal
103	SMG_RG_Mpem	Ribeira Grande	Maia | porto | Entre-marés	37°50'02''N, 25°36'47''W	Intertidal
104	SMG_RG_PFbE	Ribeira Grande	Porto Formoso | baía E	37°50'00''N, 25°33'58''W	Subtidal
105	SMG_RG_PFpb	Ribeira Grande	Porto Formoso | porto | baía	37°49'29''N, 25°34'22''W	Subtidal
106	SMG_RG_PFpem	Ribeira Grande	Porto Formoso | porto | Entre-marés	37°49'27''N, 25°34'27''W	Intertidal
107	SMG_RG_Pfprem	Ribeira Grande	Porto Formoso | praia | Entre-marés	37°49'25''N, 25°33'25''W	Intertidal
108	SMG_RG_PFpmb	Ribeira Grande	Porto Formoso | Praia dos moinhos | baía	37°50'13''N, 25°33'05''W	Subtidal
109	SMG_RG_PFsbb	Ribeira Grande	Porto Formoso | São Brás | baía	37°49'46''N, 25°35'08''W	Subtidal
110	SMG_RG_Pfztem	Ribeira Grande	Porto Formoso | Zona termal | Entre-marés	37°49'36''N, 25°31'38''W	Intertidal
111	SMG_RG_RPcem	Ribeira Grande	Rabo de Peixe | Calhetas | Entre-marés	37°49'28''N, 25°23'42''W	Intertidal
112	SMG_RG_RGbem	Ribeira Grande	Ribeira Grande | Bandejo | Entre-marés	37°49'17''N, 25°27'51''W	Intertidal
113	SMG_RG_Rfpb	Ribeira Grande	Ribeirinha | Furna da Pataca | baía	37°50'35''N, 25°30'52''W	Subtidal
114	SMG_RG_Rpcb	Ribeira Grande	Ribeirinha | Ponta do Cintrão | baía	37°50'11''N, 25°31'20''W	Subtidal
115	SMG_RG_Rpsib	Ribeira Grande	Ribeirinha | Porto de Santa Iria | baía	37°50'08''N, 25°30'59''W	Subtidal
116	SMG_RG_RpsibW	Ribeira Grande	Ribeirinha | Porto de Santa Iria | baía W	37°50'34''N, 25°30'06''W	Subtidal
117	SMG_RG_Rpsiem	Ribeira Grande	Ribeirinha | Porto de Santa Iria | Entre-marés	37°50'08''N, 25°30'56''W	Intertidal
118	SMG_PD_SVpoemw	Ribeira Grande	São Vicente | Poços | Entre-marés (W)	37°50'03''N, 25°19'53''W	Intertidal
119	SMG_VF_AA_bgs	Vila Franca do Campo	Água de Alto | Baixa da Garoupa | Subtidal	37°42'31''N, 25°31'47''W	Subtidal
120	SMG_VF_AAbr	Vila Franca do Campo	Água de Alto | Boca da Ribeira	37°42'56''N, 25°32'48''W	Intertidal
121	SMA_VF_AAtm	Vila Franca do Campo	Água de Alto | Três Marias	37°42'29''N, 25°31'56''W	Subtidal
122	SMG_VF_PGb	Vila Franca do Campo	Ponta Garça | baía	37°42'56''N, 25°36'41''W	Intertidal
123	SMG_VF_PGb	Vila Franca do Campo	Ponta Garça | baía	37°42'56''N, 25°36'41''W	Subtidal
124	SMG_VF_PGbE	Vila Franca do Campo	Ponta Garça | baía E	37°42'46''N, 25°37'36''W	Subtidal
125	SMG_VF_PGem1	Vila Franca do Campo	Ponta Garça | Entre-marés 1	37°42'58''N, 25°36'48''W	Intertidal
126	SMG_VF_PGem2	Vila Franca do Campo	Ponta Garça | Entre-marés 2	37°42'59''N, 25°36'41''W	Intertidal
127	SMG_VF_RPem	Vila Franca do Campo	Ribeira da Praia | Entre-marés	37°42'54''N, 25°34'43''W	Intertidal
128	SMG_VF_RTem	Vila Franca do Campo	Ribeira das Tainhas | Entre-marés	37°42'55''N, 25°35'52''W	Intertidal
129	SMG_VF_VFile	Vila Franca do Campo	Vila Franca do Campo | Ilhéu | lado externo	37°42'14''N, 25°33'27''W	Subtidal
130	SMG_VF_VFilep	Vila Franca do Campo	Vila Franca do Campo | Ilhéu | lado externo | picos	37°42'00''N, 25°33'22''W	Subtidal
131	SMG_VF_VFileE	Vila Franca do Campo	Vila Franca do Campo | Ilhéu | lado externo E	37°42'18''N, 25°33'32''W	Subtidal
132	SMG_VF_VFileS	Vila Franca do Campo	Vila Franca do Campo | Ilhéu | lado externo S	37°42'14''N, 25°33'20''W	Subtidal
133	SMG_VF_VFileSW	Vila Franca do Campo	Vila Franca do Campo | Ilhéu | lado externo SW	37°42'15''N, 25°33'19''W	Subtidal
134	SMG_VF_VFmem	Vila Franca do Campo	Vila Franca do Campo | marina | Entre-marés	37°42'54''N, 25°34'16''W	Intertidal
135	SMG_VF_VFmli	Vila Franca do Campo	Vila Franca do Campo | marina | lado interno	37°42'50''N, 25°34'12''W	Subtidal
136	SMG_VF_VFpbw	Vila Franca do Campo	Vila Franca do Campo | porto | baía (W)	37°42'45''N, 25°33'53''W	Subtidal
137	SMG_VF_VFpemw	Vila Franca do Campo	Vila Franca do Campo | porto | Entre-marés (W)	37°42'50''N, 25°33'58''W	Intertidal
138	SMG_VF_VFpem	Vila Franca do Campo	Vila Franca do Campo | praia | Entre-marés	37°42'59''N, 25°34'22''W	Intertidal

**Table 2.** Macroalgal species recorded from São Miguel Island, with information on relative abundance, origin and status.

**Phylum**	**Species (Accepted Name)**	**Number of records**	**Establishment means**	**Occurrence remarks**
Rhodophyta	*Acrosorium ciliolatum* (Harvey) Kylin	826	Native	
Rhodophyta	*Agardhinula browneae* (J.Agardh) De Toni	1	Native	
Rhodophyta	*Aglaothamnion bipinnatum* (P.Crouan & H.Crouan) Feldmann & G. Feldmann	24	Native	New record
Rhodophyta	*Aglaothamnion pseudobyssoides* (P.Crouan & H.Crouan) Halos	1	Native	
Rhodophyta	*Aglaothamnion tenuissimum* (Bonnemaison) Feldmann-Mazoyer	14	Uncertain	
Rhodophyta	*Ahnfeltiopsis devoniensis* (Greville) P.C.Silva & DeCew	5	Native	
Rhodophyta	*Amphiroa beauvoisii* J.V.Lamouroux	12	Native	
Rhodophyta	*Amphiroa cryptarthrodia* Zanardini	4	Native	
Rhodophyta	*Amphiroa rigida* J.V.Lamouroux	3	Native	
Rhodophyta	*Anotrichium barbatum* (C.Agardh) Nägeli	8	Native	
Rhodophyta	*Anotrichium furcellatum* (J.Agardh) Baldock	14	Uncertain	
Rhodophyta	*Anotrichium tenue* (C.Agardh) Nägeli	1	Native	
Rhodophyta	*Antithamnion cruciatum* (C.Agardh) Nägeli	11	Native	New record
Rhodophyta	*Antithamnion decipiens* (J.Agardh) Athanasiadis	1	Native	
Rhodophyta	*Antithamnion diminuatum* Wollaston	6	Introduced	
Rhodophyta	*Antithamnion hubbsii* E.Y.Dawson	7	Introduced	New record
Rhodophyta	*Antithamnionella boergesenii* (Cormaci & G.Furnari) Athanasiadis	1	Uncertain	
Rhodophyta	*Antithamnionella floccosa* (O.F.Müller) Whittick	1	Native	New record
Rhodophyta	*Antithamnionella spirographidis* (Schiffner) E.M.Wollaston	1	Introduced	New record
Rhodophyta	*Antithamnionella ternifolia* (J.D.Hooker & Harvey) Lyle	2	Introduced	
Rhodophyta	*Aphanocladia stichidiosa* (Funk) Ardré	2	Native	
Rhodophyta	*Apoglossum ruscifolium* (Turner) J.Agardh	2	Native	New record
Rhodophyta	*Asparagopsis armata* Harvey	188	Introduced	
Rhodophyta	*Asparagopsis armata* Harvey, phase Falkenbergia rufolanosa (Harvey) F.Schmitz	34	Introduced	
Rhodophyta	*Asparagopsis taxiformis* (Delile) Trevisan	154	Native	
Rhodophyta	*Asteromenia peltata* (W.R.Taylor) Huisman & A.J.K.Millar	6	Native	
Rhodophyta	*Balliella cladoderma* (Zanardini) Athanasiadis	3	Native	New record
Rhodophyta	*Bangia atropurpurea* (Mertens ex Roth) C.Agardh	7	Native	
Rhodophyta	*Bonnemaisonia asparagoides* (Woodward) C.Agardh	8	Native	
Rhodophyta	*Bonnemaisonia hamifera* Hariot	1	Introduced	
Rhodophyta	*Bornetia secundiflora* (J.Agardh) Thuret	6	Native	
Rhodophyta	*Bostrychia scorpioides* (Hudson) Montagne	1	Native	New record
Rhodophyta	*Botryocladia botryoides* (Wulfen) Feldmann	4	Native	
Rhodophyta	*Botryocladia macaronesica* Afonso-Carillo, Sobrino, Tittley & Neto	52	Macaronesian endemism	
Rhodophyta	*Callithamnion corymbosum* (Smith) Lyngbye	7	Native	
Rhodophyta	*Callithamnion granulatum* (Ducluzeau) C. Agardh	38	Native	
Rhodophyta	*Callithamnion tetragonum* (Withering) S.F.Gray	15	Native	New record
Rhodophyta	*Callithamnion tetricum* (Dillwyn) S.F.Gray	1	Native	
Rhodophyta	*Carradoriella denudata* (Dillwyn) Savoie & G.W.Saunders	26	Uncertain	
Rhodophyta	*Carradoriella elongata* (Hudson) A.M.Savoie & G.W.Saunders	1	Native	
Rhodophyta	*Catenella caespitosa* (Withering) L.M.Irvine	5	Native	
Rhodophyta	*Caulacanthus ustulatus* (Turner) Kützing	121	Uncertain	
Rhodophyta	*Centroceras clavulatum* (C.Agardh) Montagne	82	Native	
Rhodophyta	*Ceramium botryocarpum* A.W.Griffiths ex Harvey	12	Native	
Rhodophyta	*Ceramium ciliatum* (J.Ellis) Ducluzeau	25	Native	
Rhodophyta	*Ceramium cimbricum* H.E.Petersen	8	Native	New record
Rhodophyta	*Ceramium circinatum* (Kützing) J.Agardh	3	Native	
Rhodophyta	*Ceramium deslongchampsii* Chauvin ex Duby	6	Native	
Rhodophyta	*Ceramium diaphanum* (Lightfoot) Roth	9	Native	
Rhodophyta	*Ceramium echionotum* J.Agardh	10	Native	
Rhodophyta	*Ceramium pallidum* (Kützing) Maggs & Hommersand	24	Native	New record
Rhodophyta	*Ceramium secundatum* Lyngbye	19	Native	New record
Rhodophyta	*Ceramium tenuicorne* (Kützing) Waern	11	Native	New record
Rhodophyta	*Ceramium virgatum* Roth	23	Native	
Rhodophyta	*Ceratodictyon intricatum* (C.Agardh) R.E.Norris	2	Native	
Rhodophyta	*Champia parvula* (C.Agardh) Harvey	10	Native	
Rhodophyta	*Chondracanthus acicularis* (Roth) Fredericq	203	Native	
Rhodophyta	*Chondracanthus teedei* (Mertens ex Roth) Kützing	57	Native	
Rhodophyta	*Chondria capillaris* (Hudson) M.J.Wynne	2	Native	
Rhodophyta	*Chondria coerulescens* (J.Agardh) Sauvageau	29	Uncertain	
Rhodophyta	*Chondria dasyphylla* (Woodward) C.Agardh	22	Uncertain	
Rhodophyta	*Coelothrix irregularis* (Harvey) Børgesen	2	Native	
Rhodophyta	*Compsothamnion decompositum* (J.Agardh) Maggs & L'Hardy-Halos	2	Native	
Rhodophyta	*Corallina ferreyrae* E.Y.Dawson, Acleto & Foldvik	30	Native	New record
Rhodophyta	*Corallina officinalis* Linnaeus	140	Native	
Rhodophyta	*Cottoniella filamentosa* (M.Howe) Børgesen	15	Native	New record
Rhodophyta	*Crouania attenuata* (C.Agardh) J.Agardh	5	Native	
Rhodophyta	*Cruoria pellita* (Lyngbye) Fries	2	Native	New record
Rhodophyta	*Cryptonemia crenulata* (J.Agardh) J.Agardh	1	Uncertain	New record
Rhodophyta	*Cryptonemia seminervis* (C.Agardh) J.Agardh	2	Native	
Rhodophyta	*Cryptopleura ramosa* (Hudson) L.Newton	9	Native	
Rhodophyta	*Dasya baillouviana* (S.G.Gmelin) Montagne	6	Uncertain	
Rhodophyta	*Dasya caraibica* Børgesen	1	Native	New record
Rhodophyta	*Dasya corymbifera* J.Agardh	3	Native	
Rhodophyta	*Dasya crouaniana* J.Agardh	1	Native	New record
Rhodophyta	*Dasya hutchinsiae* Harvey	6	Native	
Rhodophyta	*Dasya ocellata* (Grateloup) Harvey	3	Native	
Rhodophyta	*Dermocorynus dichotomus* (J.Agardh) Gargiulo, M.Morabito & Manghisi	67	Native	
Rhodophyta	*Diplothamnion jolyi* C.Hoek	8	Native	
Rhodophyta	*Drachiella heterocarpa* (Chauvin ex Duby) Maggs & Hommersand	5	Native	New record
Rhodophyta	*Dudresnaya crassa* M.Howe	7	Native	
Rhodophyta	*Dudresnaya verticillata* (Withering) Le Jolis	2	Native	
Rhodophyta	*Ellisolandia elongata* (J.Ellis & Solander) K.R.Hind & G.W.Saunders	67	Native	
Rhodophyta	*Erythrocystis montagnei* (Derbès & Solier) P.C.Silva	4	Native	
Rhodophyta	*Erythrodermis traillii* (Holmes ex Batters) Guiry & Garbary	10	Uncertain	
Rhodophyta	*Erythroglossum laciniatum* (Lightfoot) Maggs & Hommersand	1	Native	New record
Rhodophyta	*Erythrotrichia carnea* (Dillwyn) J.Agardh	1	Uncertain	
Rhodophyta	*Eupogodon planus* (C.Agardh) Kützing	2	Native	
Rhodophyta	*Gaillona gallica* (Nägeli) Athanasiadis	19	Native	
Rhodophyta	*Gaillona hookeri* (Dillwyn) Athanasiadis	9	Native	
Rhodophyta	*Gastroclonium clavatum* (Roth) Ardissone	1	Native	
Rhodophyta	*Gastroclonium ovatum* (Hudson) Papenfuss	17	Native	
Rhodophyta	*Gastroclonium reflexum* (Chauvin) Kützing	23	Native	
Rhodophyta	*Gayliella flaccida* (Harvey ex Kützing) T.O.Cho & L.J.McIvor	8	Native	
Rhodophyta	*Gelidium corneum* (Hudson) J.V.Lamouroux	2	Native	
Rhodophyta	*Gelidium microdon* Kützing	228	Native	
Rhodophyta	*Gelidium pusillum* (Stackhouse) Le Jolis	52	Native	
Rhodophyta	*Gelidium spinosum* (S.G.Gmelin) P.C.Silva	150	Native	
Rhodophyta	*Gigartina pistillata* (S.G.Gmelin) Stackhouse	20	Native	
Rhodophyta	*Gracilaria gracilis* (Stackhouse) Steentoft, L.M.Irvine & Farnham	3	Native	New record
Rhodophyta	*Gracilaria multipartita* (Clemente) Harvey	1	Native	
Rhodophyta	*Gracilariopsis longissima* (S.G.Gmelin) Steentoft, L.M.Irvine & Farnham	12	Native	
Rhodophyta	*Grallatoria reptans* M.Howe	2	Introduced	
Rhodophyta	*Grateloupia filicina* (J.V.Lamouroux) C.Agardh	21	Native	
Rhodophyta	*Griffithsia corallinoides* (Linnaeus) Trevisan	2	Native	
Rhodophyta	*Griffithsia phyllamphora* J.Agardh	2	Uncertain	
Rhodophyta	*Gymnogongrus crenulatus* (Turner) J.Agardh	107	Native	
Rhodophyta	*Gymnogongrus griffithsiae* (Turner) C.Martius	115	Native	
Rhodophyta	*Gymnophycus hapsiphorus* Huisman & Kraft	1	Introduced	
Rhodophyta	*Gymnothamnion elegans* (Schousboe ex C.Agardh) J.Agardh	4	Native	
Rhodophyta	*Halarachnion ligulatum* (Woodward) Kützing	26	Native	
Rhodophyta	*Halurus flosculosus* (J.Ellis) Maggs & Hommersand	4	Native	
Rhodophyta	*Haraldia lenormandii* (Derbès & Solier) Feldmann	1	Native	New record
Rhodophyta	*Haraldiophyllum bonnemaisonii* (Kylin) A.D.Zinova	17	Native	New record
Rhodophyta	*Herposiphonia secunda* (C.Agardh) Ambronn	19	Native	
Rhodophyta	*Heterosiphonia crispella* (C.Agardh) M.J.Wynne	5	Native	
Rhodophyta	*Hildenbrandia rubra* (Sommerfelt) Meneghini	8	Native	
Rhodophyta	*Hypnea arbuscula* P.J.L.Dangeard	1	Native	
Rhodophyta	*Hypnea cervicornis* J.Agardh	2	Native	New record
Rhodophyta	*Hypnea musciformis* (Wulfen) J.V.Lamouroux	148	Uncertain	
Rhodophyta	*Hypoglossum hypoglossoides* (Stackhouse) Collins & Hervey	16	Native	
Rhodophyta	*Itonoa marginifera* (J.Agardh) Masuda & Guiry	5	Native	
Rhodophyta	*Jania crassa* J.V.Lamouroux	3	Native	
Rhodophyta	*Jania longifurca* Zanardini	11	Uncertain	
Rhodophyta	Jania pedunculata var. adhaerens (J.V.Lamouroux) A.S.Harvey, Woelkerling & Reviers	4	Native	
Rhodophyta	*Jania rubens* (Linnaeus) J.V.Lamouroux	14	Native	
Rhodophyta	*Jania squamata* (Linnaeus) J.H.Kim, Guiry & H.-G.Choi	2	Native	
Rhodophyta	*Jania verrucosa* J.V.Lamouroux	9	Native	
Rhodophyta	*Jania virgata* (Zanardini) Montagne	9	Uncertain	
Rhodophyta	*Kallymenia reniformis* (Turner) J.Agardh	37	Native	
Rhodophyta	*Laurencia brongniartii* J.Agardh	2	Introduced	New record
Rhodophyta	*Laurencia dendroidea* J.Agardh	1	Introduced	
Rhodophyta	*Laurencia intricata* J.V.Lamouroux	2	Native	New record
Rhodophyta	*Laurencia microcladia* Kützing	1	Native	New record
Rhodophyta	*Laurencia obtusa* (Hudson) J.V.Lamouroux	23	Native	
Rhodophyta	*Laurencia pyramidalis* Bory ex Kützing	8	Native	
Rhodophyta	*Laurencia tenera* C.K.Tseng	1	Native	
Rhodophyta	*Laurencia viridis* Gil-Rodríguez & Haroun	21	Macaronesian endemism	
Rhodophyta	*Laurenciella marilzae* (Gil-Rodríguez, Sentíes, Díaz-Larrea, Cassano & M.T.Fujii) Gil-Rodríguez, Sentíes, Díaz-Larrea, Cassano & M.T.Fujii	6	Native	New record
Rhodophyta	*Leptosiphonia brodiei* (Dillwyn) Savoie & G.W.Saunders	14	Uncertain	
Rhodophyta	*Liagora distenta* (Mertens ex Roth) J.V.Lamouroux	21	Native	New record
Rhodophyta	*Liagora viscida* (Forsskål) C.Agardh	11	Native	
Rhodophyta	*Lomentaria articulata* (Hudson) Lyngbye	128	Native	
Rhodophyta	*Meredithia microphylla* (J.Agardh) J.Agardh	55	Native	New record
Rhodophyta	*Millerella tinerfensis* (Seoane-Camba) S.M.Boo & J.M.Rico	4	Macaronesian endemism	New record
Rhodophyta	*Monosporus pedicellatus* (Smith) Solier	1	Native	
Rhodophyta	*Myriogramme minuta* Kylin	12	Native	
Rhodophyta	*Nemalion elminthoides* (Velley) Batters	41	Native	
Rhodophyta	*Neoizziella divaricata* (C.K.Tseng) S.-M.Lin, S.-Y.Yang & Huisman	14	Introduced	
Rhodophyta	*Neopyropia leucosticta* (Thuret) L.-E.Yang & J.Brodie	1	Native	New record
Rhodophyta	*Nitophyllum punctatum* (Stackhouse) Greville	19	Native	
Rhodophyta	*Osmundea hybrida* (A.P.de Candolle) K.W.Nam	12	Native	
Rhodophyta	*Osmundea oederi* (Gunnerus) G.Furnari	1	Native	
Rhodophyta	*Osmundea pinnatifida* (Hudson) Stackhouse	151	Native	
Rhodophyta	*Osmundea truncata* (Kützing) K.W.Nam & Maggs	7	Native	
Rhodophyta	*Palisada corallopsis* (Montagne) Sentíes, Fujii & Díaz-Larrea	2	Native	New record
Rhodophyta	*Peyssonnelia squamaria* (S.G.Gmelin) Decaisne ex J.Agardh	71	Native	
Rhodophyta	*Phyllophora crispa* (Hudson) P.S.Dixon	37	Native	
Rhodophyta	*Phyllophora gelidioides* P.Crouan & H.Crouan ex Karsakoff	2	Macaronesian endemism	
Rhodophyta	*Phyllophora sicula* (Kützing) Guiry & L.M.Irvine	5	Native	
Rhodophyta	*Platoma cyclocolpum* (Montagne) F.Schmitz	139	Native	
Rhodophyta	*Platysiphonia delicata* (Clemente) Cremades	3	Native	New record
Rhodophyta	*Pleonosporium borreri* (Smith) Nägeli	7	Native	
Rhodophyta	*Plocamium cartilagineum* (Linnaeus) P.S.Dixon	173	Native	
Rhodophyta	*Pneophyllum confervicola* (Kützing) Y.M.Chamberlain	1	Native	New record
Rhodophyta	*Polysiphonia atlantica* Kapraun & J.N.Norris	6	Native	
Rhodophyta	*Polysiphonia havanensis* Montagne	1	Native	
Rhodophyta	*Polysiphonia opaca* (C.Agardh) Moris & De Notaris	1	Native	
Rhodophyta	*Polysiphonia stricta* (Mertens ex Dillwyn) Greville	3	Native	
Rhodophyta	*Porphyra umbilicalis* Kützing	14	Native	
Rhodophyta	*Porphyrostromium ciliare* (Carmichael) M.J.Wynne	3	Native	
Rhodophyta	*Predaea feldmannii* Børgesen	3	Native	New record
Rhodophyta	Predaea feldmannii subsp. azorica Gabriel	4	Azorean endemism	
Rhodophyta	*Pterocladiella capillacea* (S.G.Gmelin) Santelices & Hommersand	377	Native	
Rhodophyta	*Pterothamnion crispum* (Ducluzeau) Nägeli	54	Native	
Rhodophyta	*Pterothamnion plumula* (J.Ellis) Nägeli	6	Native	
Rhodophyta	*Ptilothamnion pluma* (Dillwyn) Thuret	4	Uncertain	
Rhodophyta	*Radicilingua thysanorhizans* (Holmes) Papenfuss	8	Native	New record
Rhodophyta	*Rhodophyllis divaricata* (Stackhouse) Papenfuss	7	Native	
Rhodophyta	*Rhodymenia holmesii* Ardissone	92	Native	
Rhodophyta	*Rhodymenia pseudopalmata* (J.V.Lamouroux) P.C.Silva	18	Native	
Rhodophyta	*Scagelia pylaisaei* (Montagne) M.J.Wynne	4	Native	
Rhodophyta	*Scageliopsis patens* E.M.Wollaston	1	Introduced	
Rhodophyta	*Schimmelmannia schousboei* (J.Agardh) J.Agardh	29	Native	New record
Rhodophyta	*Schizymenia apoda* (J.Agardh) J.Agardh	89	Native	
Rhodophyta	*Schottera nicaeensis* (J.V.Lamouroux ex Duby) Guiry & Hollenberg	28	Uncertain	
Rhodophyta	*Scinaia furcellata* (Turner) J.Agardh	33	Native	
Rhodophyta	*Scinaia interrupta* (A.P.de Candolle) M.J.Wynne	34	Native	
Rhodophyta	*Sebdenia dichotoma* Berthold	19	Native	
Rhodophyta	*Sebdenia rodrigueziana* (Feldmann) Codomier ex Athanasiadis	41	Native	
Rhodophyta	*Spermothamnion repens* (Dillwyn) Magnus	5	Native	
Rhodophyta	*Sphaerococcus coronopifolius* Stackhouse	68	Native	
Rhodophyta	*Spyridia filamentosa* (Wulfen) Harvey	1	Native	
Rhodophyta	*Stichothamnion cymatophilum* Børgesen	1	Native	
Rhodophyta	*Stylonema alsidii* (Zanardini) K.M.Drew	2	Native	
Rhodophyta	*Stylonema cornu-cervi* Reinsch	3	Native	
Rhodophyta	*Symphyocladia marchantioides* (Harvey) Falkenberg	85	Introduced	
Rhodophyta	*Taenioma nanum* (Kützing) Papenfuss	1	Native	New record
Rhodophyta	*Tenarea tortuosa* (Esper) Me.Lemoine	3	Native	
Rhodophyta	*Vertebrata foetidissima* (Cocks ex Bornet) Díaz-Tapia & Maggs	1	Native	
Rhodophyta	*Vertebrata fruticulosa* (Wulfen) Kuntze	5	Native	
Rhodophyta	*Vertebrata fucoides* (Hudson) Kuntze	7	Uncertain	
Rhodophyta	*Vertebrata furcellata* (C.Agardh) Kuntze	6	Native	
Rhodophyta	*Vertebrata hypnoides* (Welwitsch) Kuntze	4	Uncertain	
Rhodophyta	*Vertebrata nigra* (Hudson) Díaz-Tapia & Maggs	1	Native	New record
Rhodophyta	*Vertebrata reptabunda* (Suhr) Díaz-Tapia & Maggs	4	Uncertain	
Rhodophyta	*Vertebrata tripinnata* (Harvey) Kuntze	3	Native	
Rhodophyta	*Wrangelia penicillata* (C.Agardh) C.Agardh	6	Native	New record
Rhodophyta	*Wurdemannia miniata* (Sprengel) Feldmann & Hamel	1	Native	
Rhodophyta	*Xiphosiphonia ardreana* (Maggs & Hommersand) Savoie & G.W.Saunders	4	Native	
Rhodophyta	*Xiphosiphonia pennata* (C.Agardh) Savoie & G.W.Saunders	2	Introduced	
Rhodophyta	*Xiphosiphonia pinnulata* (Kützing) Savoie & G.W.Saunders	2	Introduced	
Chlorophyta	*Anadyomene stellata* (Wulfen) C.Agardh	4	Uncertain	
Chlorophyta	*Blidingia marginata* (J.Agardh) P.J.L.Dangeard ex Bliding	1	Native	New record
Chlorophyta	*Blidingia minima* (Nägeli ex Kützing) Kylin	2	Native	
Chlorophyta	*Bryopsis cupressina* J.V.Lamouroux	27	Native	
Chlorophyta	*Bryopsis duplex* De Notaris	1	Native	
Chlorophyta	*Bryopsis hypnoides* J.V.Lamouroux	28	Native	
Chlorophyta	*Bryopsis pennata* J.V.Lamouroux	6	Native	New record
Chlorophyta	*Bryopsis plumosa* (Hudson) C.Agardh	71	Native	
Chlorophyta	*Caulerpa prolifera* (Forsskål) J.V.Lamouroux	10	Introduced	
Chlorophyta	*Chaetomorpha aerea* (Dillwyn) Kützing	10	Native	
Chlorophyta	*Chaetomorpha linum* (O.F.Müller) Kützing	26	Native	
Chlorophyta	*Chaetomorpha pachynema* (Montagne) Kützing	2	Native	
Chlorophyta	*Cladophora albida* (Nees) Kutzing	5	Native	
Chlorophyta	*Cladophora coelothrix* Kützing	15	Native	
Chlorophyta	*Cladophora conferta* P.Crouan & H.Crouan	5	Native	
Chlorophyta	*Cladophora dalmatica* Kützing	1	Uncertain	
Chlorophyta	*Cladophora hutchinsiae* (Dillwyn) Kützing	1	Native	
Chlorophyta	*Cladophora laetevirens* (Dillwyn) Kützing	7	Uncertain	
Chlorophyta	*Cladophora lehmanniana* (Lindenberg) Kützing	5	Native	
Chlorophyta	*Cladophora liebetruthii* Grunow	1	Native	New record
Chlorophyta	*Cladophora prolifera* (Roth) Kützing	82	Native	
Chlorophyta	*Cladophoropsis membranacea* (Hofman Bang ex C.Agardh) Børgesen	1	Uncertain	
Chlorophyta	*Codium adhaerens* C. Agradh	115	Native	
Chlorophyta	*Codium decorticatum* (Woodward) M.Howe	10	Native	
Chlorophyta	*Codium elisabethiae* O.C.Schmidt	84	Macaronesian endemism	
Chlorophyta	*Codium fragile* (Suringar) Hariot	9	Native	
Chlorophyta	Codium fragile subsp. atlanticum (A.D.Cotton) P.C.Silva	3	Native	New record
Chlorophyta	Codium fragile subsp. fragile (Suringar) Hariot	15	Introduced	
Chlorophyta	*Codium tomentosum* Stackhouse	2	Native	New record
Chlorophyta	*Codium vermilara* (Olivi) Delle Chiaje	2	Native	New record
Chlorophyta	*Gayralia oxysperma* (Kützing) K.L.Vinogradova ex Scagel & *al*.	5	Native	
Chlorophyta	*Lychaete pellucida* (Hudson) M.J.Wynne	3	Native	
Chlorophyta	*Microdictyon boergesenii* Setchell	3	Native	New record
Chlorophyta	*Microdictyon umbilicatum* (Velley) Zanardini	3	Native	New record
Chlorophyta	*Pseudochlorodesmis furcellata* (Zanardini) Børgesen	1	Native	New record
Chlorophyta	*Pseudorhizoclonium africanum* (Kützing) Boedeker	5	Native	
Chlorophyta	*Ulothrix flacca* (Dillwyn) Thuret	2	Native	
Chlorophyta	*Ulva clathrata* (Roth) C.Agardh	12	Native	
Chlorophyta	*Ulva compressa* Linnaeus	12	Native	
Chlorophyta	*Ulva intestinalis* Linnaeus	37	Native	
Chlorophyta	*Ulva lactuca* Linnaeus	2	Uncertain	
Chlorophyta	*Ulva linza* Linnaeus	6	Native	
Chlorophyta	*Ulva polyclada* Kraft	1	Native	
Chlorophyta	*Ulva prolifera* O.F.Müller	6	Native	
Chlorophyta	*Ulva ralfsii* (Harvey) Le Jolis	1	Native	
Chlorophyta	*Ulva rigida* C.Agardh	198	Native	
Chlorophyta	*Valonia macrophysa* Kützing	3	Native	
Chlorophyta	*Valonia utricularis* (Roth) C. Agardh	7	Native	
Ochrophyta	*Ascophyllum nodosum* (Linnaeus) Le Jolis	8	Native	
Ochrophyta	*Bachelotia antillarum* (Grunow) Gerloff	2	Native	
Ochrophyta	*Canistrocarpus cervicornis* (Kützing) De Paula & De Clerck	2	Native	New record
Ochrophyta	*Carpomitra costata* (Stackhouse) Batters	16	Native	
Ochrophyta	*Cladostephus spongiosus* (Hudson) C.Agardh	33	Native	
Ochrophyta	*Colpomenia sinuosa* (Mertens ex Roth) Derbès & Solier	472	Native	
Ochrophyta	*Compsonema saxicola* (Kuckuck) Kuckuck	28	Native	
Ochrophyta	*Cutleria multifida* (Turner) Greville	27	Uncertain	
Ochrophyta	*Cutleria multifida* (Turner) Greville phase Aglaozonia parvula (Greville) Zanardini	4	Uncertain	
Ochrophyta	*Cystoseira compressa* (Esper) Gerloff & Nizamuddin	81	Native	
Ochrophyta	*Cystoseira foeniculacea* (Linnaeus) Greville	46	Native	
Ochrophyta	*Cystoseira humilis* Schousboe ex Kützing	21	Native	
Ochrophyta	*Dictyopteris polypodioides* (A.P.De Candolle) J.V.Lamouroux	21	Native	
Ochrophyta	*Dictyota bartayresiana* J.V.Lamouroux	9	Native	
Ochrophyta	*Dictyota ciliolata* Sonder ex Kützing	7	Native	New record
Ochrophyta	*Dictyota cyanoloma* Tronholm, De Clerck, A.Gómez-Garreta & Rull Lluch	7	Native	
Ochrophyta	*Dictyota dichotoma* (Hudson) J.V.Lamouroux	189	Native	
Ochrophyta	Dictyota dichotoma var. intricata (C.Agardh) Greville	10	Native	New record
Ochrophyta	*Dictyota fasciola* (Roth) J.V.Lamouroux	5	Native	New record
Ochrophyta	*Dictyota implexa* (Desfontaines) J.V.Lamouroux	9	Native	New record
Ochrophyta	*Ectocarpus fasciculatus* Harvey	7	Native	
Ochrophyta	*Ectocarpus siliculosus* (Dillwyn) Lyngbye	6	Uncertain	
Ochrophyta	*Elachista flaccida* (Dillwyn) Fries	1	Native	New record
Ochrophyta	*Feldmannia irregularis* (Kützing) Hamel	1	Native	
Ochrophyta	*Feldmannia mitchelliae* (Harvey) H.-S.Kim	10	Native	
Ochrophyta	*Feldmannia paradoxa* (Montagne) Hamel	3	Native	
Ochrophyta	*Fucus spiralis* Linnaeus	89	Uncertain	
Ochrophyta	*Gongolaria abies-marina* (S.G.Gmelin) Kuntze	265	Native	
Ochrophyta	*Halopteris filicina* (Grateloup) Kützing	217	Native	
Ochrophyta	*Halopteris scoparia* (Linnaeus) Sauvageau	207	Native	
Ochrophyta	*Hapalospongidion macrocarpum* (Feldmann) León-Álvarez & González-González	16	Native	New record
Ochrophyta	*Hecatonema terminale* (Kützing) Kylin	20	Native	
Ochrophyta	*Hincksia ovata* (Kjellman) P.C.Silva	4	Native	
Ochrophyta	*Hydroclathrus clathratus* (C.Agardh) M.Howe	111	Native	
Ochrophyta	*Leathesia marina* (Lyngbye) Decaisne	6	Uncertain	
Ochrophyta	*Lobophora variegata* (J.V.Lamouroux) Womersley ex E.C.Oliveira	88	Native	
Ochrophyta	*Mesogloia vermiculata* (Smith) S.F.Gray	9	Native	New record
Ochrophyta	*Myriactula rivulariae* (Suhr ex Areschoug) Feldmann	20	Native	
Ochrophyta	*Myrionema strangulans* Greville	13	Native	New record
Ochrophyta	*Nemoderma tingitanum* Schousboe ex Bornet	50	Native	
Ochrophyta	*Padina pavonica* (Linnaeus) Thivy	231	Native	
Ochrophyta	*Papenfussiella kuromo* (Yendo) Inagaki	11	Introduced	
Ochrophyta	*Petalonia binghamiae* (J.Agardh) K.L.Vinogradova	177	Introduced	
Ochrophyta	*Petalonia fascia* (O.F.Müller) Kuntze	3	Native	
Ochrophyta	*Petrospongium berkeleyi* (Greville) Nägeli ex Kützing	12	Native	
Ochrophyta	*Pseudolithoderma adriaticum* (Hauck) Verlaque	1	Native	
Ochrophyta	*Pseudolithoderma roscoffense* Loiseaux	48	Native	
Ochrophyta	*Punctaria tenuissima* (C.Agardh) Greville	4	Native	
Ochrophyta	*Ralfsia verrucosa* (Areschoug) Areschoug	92	Native	
Ochrophyta	*Sargassum cymosum* C.Agardh	20	Native	
Ochrophyta	*Sargassum furcatum* Kützing	37	Native	
Ochrophyta	*Sargassum vulgare* C.Agardh	15	Native	
Ochrophyta	*Scytosiphon lomentaria* (Lyngbye) Link	127	Native	
Ochrophyta	*Scytosiphon lomentaria* (Lyngbye) Link, phase Microspongium gelatinosum Reinke	43	Native	
Ochrophyta	*Sphacelaria cirrosa* (Roth) C.Agardh	7	Native	
Ochrophyta	*Sphacelaria plumula* Zanardini	2	Native	
Ochrophyta	*Sphacelaria rigidula* Kützing	1	Native	
Ochrophyta	*Sphacelaria tribuloides* Meneghini	5	Uncertain	
Ochrophyta	*Sphaerotrichia divaricata* (C.Agardh) Kylin	41	Uncertain	
Ochrophyta	*Sporochnus pedunculatus* (Hudson) C.Agardh	2	Native	
Ochrophyta	*Stragularia clavata* (Harvey) Hamel	1	Native	New record
Ochrophyta	*Stypopodium zonale* (J.V.Lamouroux) Papenfuss	8	Native	
Ochrophyta	*Taonia atomaria* (Woodward) J.Agardh	41	Native	
Ochrophyta	*Zanardinia typus* (Nardo) P.C.Silva	7	Native	
Ochrophyta	*Zonaria tournefortii* (J.V.Lamouroux) Montagne	248	Native	

**Table 3.** Summary of the macroalgal flora of the Island of São Miguel (N spec- number of specimens; N taxa- taxa; N spp- number of species) with information on the species origin and status (Introd- introduced; Uncrt- uncertain origin; Azo end- Azores endemism; Mac end- Macaronesia endemism; New rec- new record).

Phyllum	Order	Family	N spec	N taxa	N spp	Native	Introd	Uncrt	Azo end	Mac end	New rec
Rhodophyta	20	50	7510	284	212	171	15	21	1	4	42
Chlorophyta	5	14	1103	59	48	40	2	5		1	9
Ochrophyta	11	19	4168	88	63	55	2	6			10
Total	36	83	12781	431	323	266	19	32	1	5	61

**Table 4.** Number of macroalgae species on the Azorean Islands: Santa Maria (Neto et al. 2020d); São Miguel (the present paper); Terceira (Neto et al. 2020a); Graciosa (Neto et al. 2020c); São Jorge and Faial (authors' unpublished data); Pico (Neto et al. 2020b); Flores and Corvo (Neto et al. 2021a).

Phyllum	Santa Maria	São Miguel	Terceira	Graciosa	São Jorge	Pico	Faial	Flores	Corvo
Rhodophyta	103	212	73	126	35	142	59	80	22
Chlorophyta	29	48	24	31	17	41	16	22	8
Ochrophyta	44	63	16	38	10	42	8	26	13
Total	176	323	113	195	62	225	83	128	43
